# A Trade‐Off Between Antimicrobial Peptide Resistance and Sensitivity to Host Immune Effectors in *Staphylococcus aureus* In Vivo

**DOI:** 10.1111/eva.70068

**Published:** 2025-02-06

**Authors:** Baydaa El Shazely, Jens Rolff

**Affiliations:** ^1^ Institut für Biologie, Evolutionary Biology Freie Universität Berlin Berlin Germany; ^2^ Zoology Department, Faculty of Science Alexandria University Alexandria Egypt; ^3^ Berlin‐Brandenburg Institute of Advanced Biodiversity Research (BBIB) Berlin Germany

**Keywords:** antimicrobial peptides, antimicrobial resistance, negative pleiotropy, *Tenebrio molitor*

## Abstract

Antimicrobial peptides (AMPs) are essential immune effectors of multicellular organisms. Bacteria can evolve resistance to AMPs. Surprisingly, when used to challenge the yellow mealworm beetle, *Tenebrio molitor* , *Staphylococcus aureus* resistant to an abundant AMP (tenecin 1) of the very same host species did not increase host mortality or bacterial load compared to infections with wild‐type *S. aureus* . A possible explanation is that antimicrobial resistance is costly due to the collaterally increased sensitivity of AMP‐resistant strains to other immune effectors. Here, we study the sensitivity of a group of AMP‐resistant *S. aureus* strains (resistant to tenecin 1 or a combination of tenecin 1 + 2) to other immune effectors such as phenoloxidase and other AMPs in vivo. Using RNAi‐based knockdown, we investigate *S. aureus* survival in insect hosts lacking selected immune effectors. We find that all except one AMP‐resistant strain displayed collateral sensitivity toward phenoloxidase. Some AMP‐resistant strains show sensitivity to components of the yellow mealworm beetle AMP defense cocktail. Our findings are consistent with the idea that resistance to AMPs does not translate into changes in virulence because it is balanced by the collaterally increased sensitivity to other host immune effectors. AMP resistance fails to provide a net survival advantage to *S. aureus* in a host environment that is dominated by AMPs.

## 1 Introduction

An infection is a stepwise process where a pathogen has either to overcome different layers of immune defense or to evade recognition (Hall, Bento, and Ebert 2017). One of the first lines of defense in multicellular organisms are antimicrobial peptides (AMPs) (Lazzaro, Zasloff, and Rolff 2020). Either already constitutively expressed or induced upon infection, organisms react with an AMP defence cocktail with broad antibacterial and antifungal activities (Hanson, Kondo, and Lemaitre 2019; Johnston, Makarova, and Rolff 2014; Mylonakis et al. 2016). A reasonable assumption therefore is that AMP resistance of pathogens plays a significant role in the infection process (Cheung et al. 2018; Koprivnjak and Peschel 2011).

It is uncertain, however, whether AMP resistance is an important feature of invading pathogens. Mechanisms of bacterial evolution of resistance to AMPs are conserved (Joo, Fu, and Otto 2016) and include alteration of bacterial cell surface charge or components (Ahmad, Majaz, and Nouroz 2020; Andersson, Hughes, and Kubicek‐Sutherland 2016; Arii et al. 2019; Cheung et al. 2018). A study in mice found that AMP resistance is prerequisite for infection establishment of *Staphylococcus aureus* (Cheung et al. 2018), a major human pathogen. By contrast, we reported earlier that *S. aureus* experimentally evolved to be resistant to host and non‐host AMPs mostly does not show increased survival or virulence in the insect infection model *Tenebrio molitor* (Dobson, Purves, and Rolff 2014; El Shazely et al. 2019).

In vivo, a pathogen faces a multitude of immune effectors, which include in the case of insects, multiple effectors such as AMPs, ROS, phagocytes, and phenoloxidase. Upon infection, phagocytosis and phenoloxidase are fast acting (Haine et al. 2008). Phenoloxidase is the enzyme that drives the melanization process including encapsulation and nodulation of invading pathogens and parasites. During the production of melanin, highly toxic intermediate products such as quinones and ROS are produced, which contribute to the killing of pathogens (Keehnen et al. 2017). In contrast to the fast acting effectors, the expression of AMPs is usually ramped up hours after infection; in the case of our model *T. molitor*, it takes more than 6 h (Johnston, Makarova, and Rolff 2014).

Given the complex environment an infectious agent faces in vivo, one possible explanation for the mismatch between evolved AMP resistance and low virulence and survivorship within the host discussed above could be the collaterally increased sensitivity against components of the host defense cocktail, i.e., a trade‐off between resistance to different immune effectors. Collateral sensitivity is usually defined as the probability of evolution of sensitivity to drug A, upon acquiring resistance to drug B (Barbosa et al. 2019), of AMP‐resistant strains to other immune effectors via negative pleiotropy. Collateral sensitivity was first described in the 1950s for antibiotics (Szybalski and Bryson 1952). In our infection model *T. molitor* , it seems that some of these resistance mechanisms are pleiotropic and provide *S. aureus* with phagocytosis resistance (El Shazely et al. 2019), but this does not result in a higher bacterial load of infected beetles.

Here, we investigate whether resistance to particular host AMPs or phenoloxidase results in enhanced sensitivity to other immune effectors including other AMPs in vivo. Here, we focus on in vivo investigations as the in vivo activity of AMPs cannot be necessarily predicted from in vitro activities (Keshavarz, Zanchi, and Rolff 2023; Zanchi, Johnston, and Rolff 2017). To test our hypothesis about collateral sensitivity in vivo, we use the mealworm beetle *T. molitor* as a host and make use of a suite of *S. aureus* strains that have been evolved previously (Makarova et al. 2016). These strains were selected for resistance to either the *T. molitor* AMP tenecin 1 or both tenecin 1 and tenecin 2. It was previously reported that resistance to these AMPs was associated with mutations in either the *pmt*RS or *nsa*RS operons (Table S1), which encodes an export system for cellular toxins called phenol‐soluble modulin (Chatterjee et al. 2013) and a nisin susceptibility–associated (*nsa*) system (Hiron et al. 2011), respectively. These two‐component system operons are involved in envelope stress tolerance (Johnston, Dobson, and Rolff 2016; Makarova et al. 2018). A second resistance mutation was found in some strains in the *rpo*BC operon (Table S1) coding for the RNA polymerase subunits β and β′, which increased the bacterial lag phase in *nsa* mutants but not in *pmt* mutants (Makarova et al. 2018) and is reported as a complementary mutation to antibiotic resistance in other species (Cho and Misra 2021).

We hypothesized that AMP‐resistant pathogens pay a cost by evolving sensitivity to other immune effectors such as other AMPs and phenoloxidase, a fast‐reacting crucial part of the arthropod immune system that melanizes pathogens and generates cytotoxic substances (Dudzic et al. 2015). We studied if collateral sensitivity of AMP‐resistant *S. aureus* from our previous work (Makarova et al. 2018) to other immune effectors in vivo is mutation‐dependent. To test this hypothesis, we assessed the infection dynamics of *S. aureus* over 7 days in insect hosts where we knocked down selected immune effectors using RNAi. We performed RNAi‐based knockdown of either prophenoloxidase (PPO) or a group of selected AMP genes in *T. molitor* . An earlier RNAseq study from our laboratory showed that an array of AMPs is upregulated in *T. molitor* upon *S. aureus* infection, including one defensin, three coleoptercins, and four attacins (Johnston, Makarova, and Rolff 2014). Thus, we knocked down the combination of three of the most highly expressed AMP genes, which are the defensin tenecin 1, the coleoptercin tenecin 2, and the attacin tenecin 4. Tenecin 1 is primarily directed against Gram‐positive bacteria (Moon et al. 1994), while the other two AMPs showed very limited activity toward *S. aureus* in vitro (Chae et al. 2012; Roh et al. 2009). Knockdowns of tenecin 2 and 4, however, resulted in a reduced host survival upon *S. aureus* infection with an AMP‐sensitive *S. aureus* strain (Zanchi, Johnston, and Rolff 2017), demonstrating an in vivo activity of these two AMPs. We quantified bacterial load as a measure of bacterial survival and hence bacterial fitness (Dobson, Purves, and Rolff 2014; McGonigle, Purves, and Rolff 2016) and reduced host survival as a measure of virulence (Zanchi, Johnston, and Rolff 2017).

## 2 Materials and Methods

### 2.1 Rearing of *Tenebrio molitor*

The mealworm beetles, *Tenebrio molitor*, were reared as previously described (El Shazely et al. 2019). Experiments were performed on 7–9‐day‐old females with a weight ranging between 0.120 and 0.190 g (see the Appendix S1).

### 2.2 Gene Knockdown by RNA Interference

A double‐stranded RNA injection was used to knockdown gene expression by RNA interference as described by Zanchi, Johnston, and Rolff (2017) and Khan, Agashe, and Rolff (2017). Briefly, we used synthetic constructs as templates for tenecin 1, tenecin 2, and tenecin 4 dsRNA syntheses. At least two PPO genes were reported, previously, in a *T. molitor* RNAseq study (Johnston, Makarova, and Rolff 2014), which encode two subunits of a heterodimer enzyme. We synthesized PPO RNAi using the internal region of *T. molitor* cDNA as a template for PO1 and PO2 dsRNA as described previously (Khan, Prakash, and Agashe 2017). We used RNAi based on *Galleria mellonella* lysozyme (Lys) as a procedural control because it has no proven homology of sequence with any known gene of *T. molitor* (Johnston and Rolff 2015; Zanchi, Johnston, and Rolff 2017).

Templates were amplified by PCR (KAPA2G Fast ReadyMix, KAPA Biosystems) using gene‐specific primers (Table S2) tailed with the T7 polymerase promoter sequence (Sigma‐Aldrich). Integrity and length of the amplicon were checked by running it on a 2% agarose gel and cleaned up using a kit (PCR DNA Clean‐Up Kit, Roboklon). The resulting amplicon was used as a template for RNA synthesis (T7 RiboMAX Express Large Scale RNA Production System, Promega) according to the manufacturer's recommendations. We then purified the RNA with a phenol–chloroform extraction. Finally, the RNA pellet was resuspended in a nuclease‐free insect ringer solution (128 mM NaCl, 18 mM CaCl_2_, 1.3 mM KCl, 2.3 mM) and kept in −80°C freezer. To obtain dsRNA, annealing was performed by warming up to 65°C for 30 min and then allowing to gradually cool down and incubated at least 15 min at 22°C. A quantitative real‐time PCR was carried out to check the efficiency of gene knockdown as described in the Appendix S1.

### 2.3 Experimental Design

We performed two knockdown experiments, either of PPO Figure S1 (PO1 and PO2, previously described in Khan, Prakash, and Agashe (2017)) or of a three‐component AMP defence cocktail Figure S2 (T1T2T4) as follows.

#### 2.3.1 Experiment 1: Collateral Sensitivity of Tenecin‐Resistant Pathogen ( *S. aureus* ) Toward Phenoloxidase

An overview of this experiment is provided in Figure S1. To perform PO1 and PO2 knockdown (PO KD) of *T. molitor* PPO, we injected 2100 ± 100 ng dsRNA at a concentration of ca. 350 ng/μL in a 6‐μL insect ringer solution, containing equal quantities of dsPO1 and dsPO2. The procedural control (Lys) received the same dsRNA concentration and total injection volume. Because the PPO gene is constitutively expressed, it is necessary to deplete PO that had already been present in the hemolymph before the knockdown (Khan, Prakash, and Agashe 2017); therefore, we injected each beetle with 5‐μL peptidoglycan (Sigma‐Aldrich, Cat # 77140, concentration: 100 ng/mL suspended in ringer solution) 2 days after RNAi manipulation. Two days later, *T. molitor* was challenged with either the tenecin‐sensitive or ‐resistant *S. aureus* strain, listed in Table 1. Results of the relative expression analysis of PPO gene in beetles with different knockdown treatments are shown in Figure S3.

**TABLE 1:** The AMP‐selected *Staphylococcus aureus* strains and their corresponding controls (nested per line; Makarova et al. 2018) used in this study.

Strain	Mutations	Tenecin sensitivity/resistance	Line
Ancestor (SH1000)	None	Sensitive strain	—
C1	None	Sensitive strain	1
T1‐1L	*rpo*	T1‐resistant strain	1
C2	None	Sensitive strain	2
T1‐2L	*pmt*	T1‐resistant strain	2
T1‐2S	*pmt‐rpo*	T1‐resistant strain	2
T1T2‐2	*nsa*	T1T2‐resistant strain	2
C3	None	Sensitive strain	3
T1T2‐3	*nsa‐rpo*	T1T2‐resistant strain	3

*Note:* All strains had originally experimentally evolved in the presence of either tenecin 1 or tenecin 1 plus tenecin 2. *S. aureus* SH1000 is the tenecin‐sensitive ancestor strain. C1, C2, and C3 are the passaged unselected *S. aureus* strains (procedural controls) of lines 1, 2, and 3, respectively. T1‐1L, T1‐2S, and T1‐2L are tenecin 1‐selected *S. aureus* strains, the first strain belongs to line 1 with large‐shaped colonies, while the other two strains belong to line 2 with small‐ and large‐shaped colonies, respectively. T1T2‐2, T1T2‐3 are the tenecin 1 plus tenecin 2‐selected *S. aureus* strains, the first belongs to line 2, while the latter belongs to line 3.

#### 2.3.2 Experiment 2: Collateral Sensitivity of T1‐ and T1T2‐Resistant *S. aureus* Toward T2 and/or T4 Knockdowns (AMPs of the Defence Cocktail)

An overview of this experiment is provided in Figure S2. In the case of T1T2T4 knockdowns, we injected 3000 ± 100 ng dsRNA in a 6‐μL total volume of ringer (i.e., circa 1000 ng for each gene), resulting in a concentration of 500 ng/μL of dsRNA in 2 μL of ringer solution for each gene. The procedural control (Lys) received the same dsRNA concentration and total injection volume. Female insects were infected with one of AMP‐sensitive or ‐resistant *S. aureus* strains 2 days later. Results of the relative expression analysis of the tenecin 1, tenecin 2, and tenecin 4 genes in beetles with different knockdown treatments are shown in Figure S4.

It should be noted that the procedural controls from the two experiments differ. Procedural controls of the PO knockdown experiment were injected with 2100 ± 100 ng dsRNA in a 5‐μL total volume of ringer and those of AMPS (T1T2T4) were injected with 3000 ± 100 ng dsRNA in a 6‐μL total volume of ringer. All beetles in the PO kd treatment received an extra injection prior to the KD treatment to deplete constitutive phenoloxidase (see Khan, Agashe, and Rolff 2017 and references therein).

#### 2.3.3 Bacterial Strains

We picked five AMP‐resistant *Staphylococcus aureus* strains, their three respective sensitive controls, and the ancestor strain (SH1000) that was used in our original work to evolve the AMP‐resistant lines (Table 1, described previously, MICs of Tenebrio AMPs are reported in this paper, Makarova et al. 2018). The protocol to generate those strains is described here in short (more detail in Makarova et al. 2018). Five parallel lines derived from individual clones of *S. aureus* SH1000 were used to start five independent selection lines. These five clones are the five ancestors. Serial passage of bacteria started at 0.5 of the MIC for either tenecin 1 or tenecin 1 + 2. We also ran controls in a culture medium simultaneously. The whole selection lasted for 7 days, and the AMP concentrations were doubled daily. All lines were able to grow in the presence of 256 μg/mL of tenecin 1 and 512 μg/mL of tenecin 2, resulting in an increase in MIC for tenecin 1. The ancestor strains showed MICs of 4 μg/mL for tenecin 1 and 4 μg/mL of tenecin 1 and 8 μg/mL of tenecin 2 for the combined treatment. For the experiments carried out here, we chose a subset of selection lines and their respective controls (see Table 1). The AMP‐resistant strains are therefore either highly resistant to tenecin 1 or a combination of tenecin 1 plus tenecin 2. AMP‐resistant strains are representative of the five resistance mutations that we studied previously (El Shazely et al. 2019). We found that our experimentally evolved AMP‐resistant *S. aureus* strains harbored either of the following mutations in the *pmt*, *nsa*, *rpo*, *pmt‐rpo* or *nsa‐rpo* operons (listed in Table 1). All strains were kept as glycerol stocks in −80°C prior to use.

#### 2.3.4 Bacterial Culture and Injection

*S. aureus* was streaked over Müller Hinton (MH) agar from a glycerol stock and incubated at 30°C for 48 h. A liquid culture was obtained by scratching three separate colonies with a culture needle and transferring to MH broth (PanReac AppliChem, Cat # 413788.1210) and then incubated overnight at 25°C as previously described (Zanchi, Johnston, and Rolff 2017). The bacterial culture was washed three times by centrifuging for 10 min at 7500 *g*. The supernatant was discarded, and the pellet was resuspended in a ringer solution (Sigma‐Aldrich, Cat # 96724‐100TAB). Each female beetle received 5 μL of the prepared inoculum, which was standardized to a bacterial load of 6 × 10^6^ CFU (colony‐forming units).

A manual injector attached to a sterile disposable capillary needle was used to inject into the intersegmental membrane of the insect, between the fourth and fifth abdominal sternites, parallel to the anterior–posterior axis of the body. Injections were alternated between the left and right sides of the beetles for dsRNA, peptidoglycan (if used), and bacterial injections to avoid multiple wounding at the same spot. The specimen was discarded and replaced if leakage was noticed. The negative control group (sham‐infected) was injected with a 5‐μL ringer solution.

### 2.4 Quantification of Infection‐Bacterial Survival Inside the Host

Colony‐forming units (CFUs) were monitored at 1‐ and 7‐days post‐infection consistent with previous findings on the temporal dynamics of *S. aureus* in *T. molitor* (El Shazely et al. 2019; Zanchi, Johnston, and Rolff 2017). Sampling bacterial load at a time point later than 10‐days post‐infection was not feasible due to the substantial host mortality rate. *Staphylococcus aureus* was recovered by a perfusion bleed as previously described (El Shazely et al. 2019; Haine et al. 2008). Each group consisted of 15 beetles, divided into three replicates. The beetles were placed over ice for 10–15 min. They were washed with 70% ethyl alcohol and carefully dried. After making a small incision in the abdominal lateral periphery, 500 μL of phosphate buffer saline (PBS, Chem Solute, Cat # 8435.0100) was injected into the hemocoel using a 22‐gauge needle (Henke Sass Wolf FINE‐JECT, Cat # 4710004020) that was inserted between the head and thorax. Then, from the abdominal incision, the outflow was collected into a sterile 1.5‐mL tube. The collected hemolymph was vortexed, and then 100 μL was plated on MH agar. We performed serial dilution of the hemolymph. CFUs were counted manually 2 days after the plates were incubated at 30°C.

### 2.5 Host Survival

We also checked whether the virulence of AMP‐resistant strains as inferred from the survival of *T. molitor* is controlled by the host phenoloxidase and other components of the AMP defence cocktail. Each group was injected with one of nine *S. aureus* strains (Figures S1 and S2). Each group consisted of 30 beetles divided into three replicates. Negative control was injected with ringer solution (sham‐infected). The study only included female beetles. Mortality was monitored for 30‐days post‐injection.

### 2.6 Data Analysis

All the data were analyzed in R version 4.4.0 [40].

#### 2.6.1 Quantification of Infection in *T. molitor* With Different Knockdown Treatments

The sham‐infected group showed no recovered *S. aureus* CFU. Thus, it was excluded from further analysis. First, we analyzed whether the sensitivity of particular AMP‐resistant strains toward knockdown of PPO and a cocktail of AMPs T1T2T4 could explain the variations in the number of CFU of the recovered *S. aureus* strains at two time points of infection (1‐ and 7‐days post‐infection). To account for the nested ontogeny of the nine *S. aureus* strains (Table 1), a mixed model was fitted to the dataset, and “lines” was implemented as a random factor. The CFU data were best fitted when Box–Cox transformed (power transform).

We used the function “lme” from “nlme” package to run a linear mixed model on the Box–Cox transformed data. The heteroscedasticity of the variances between each knockdown treatment was modeled using the function “varIdent” implemented in the weight of the lmm model. *Post hoc* comparisons were performed using “lsmeans” with a “FDR” adjustment from package “lsmeans”. Models were assessed and compared using “Anova” function from package “car”.

#### 2.6.2 Host Survival Analysis

We tested whether the AMP resistance of the infecting strains and knockdown treatment of the beetles as well as the interaction between them explained the mortality of the beetles over 30 days. Based on the assumption of nonconstant hazard using Weibull errors, we analyzed the data using the “survreg” function from the “survival” package. Post hoc comparisons were performed using “pairwise_survdiff” from package “survminer” with the “Benjamini–Hochberg” adjustment.

## 3 Results

We first studied the bacterial load in beetles injected with the ancestral strain of *S. aureus* from our panel (SH1000) and then beetle mortality when either phenoloxidase or the combination of tenecins 1, 2, and 4 were knocked down. We then studied in the same way the following strains with the prediction that they would have higher bacterial loads in beetles where either phenoloxidase or the combination of tenecins 1, 2, and 4 were knocked down and that these knockdown beetles would suffer higher mortalities compared to injections with the ancestral strain. We tested a tenecin 1‐resistant *rpo S. aureus* mutant; *rpo* is a gene that encodes an RNA polymerase subunit and is known to increase AMP and rifampin resistance (El Shazely et al. 2020; Mariam et al. 2004). We then tested strains with mutations in *pmt* or *nsa* operons or double mutations in either *pmt‐rpo* or *nsa‐rpo*. *Pmt* is related to envelope stress responses; *nsa* codes for a nisin‐associated susceptibility two‐component system (Yoshida et al. 2011). The mortality rate and the bacterial load of AMP‐resistant mutants were compared to their respective sensitive procedural controls.

### 3.1 The Knockdown of Phenoloxidase or the AMP Combination Did Not Affect the Bacterial Load of *Staphylococcus aureus* SH1000 (Ancestor Strain) but Increased the Mortality of Infected Hosts

**FIGURE 1:**
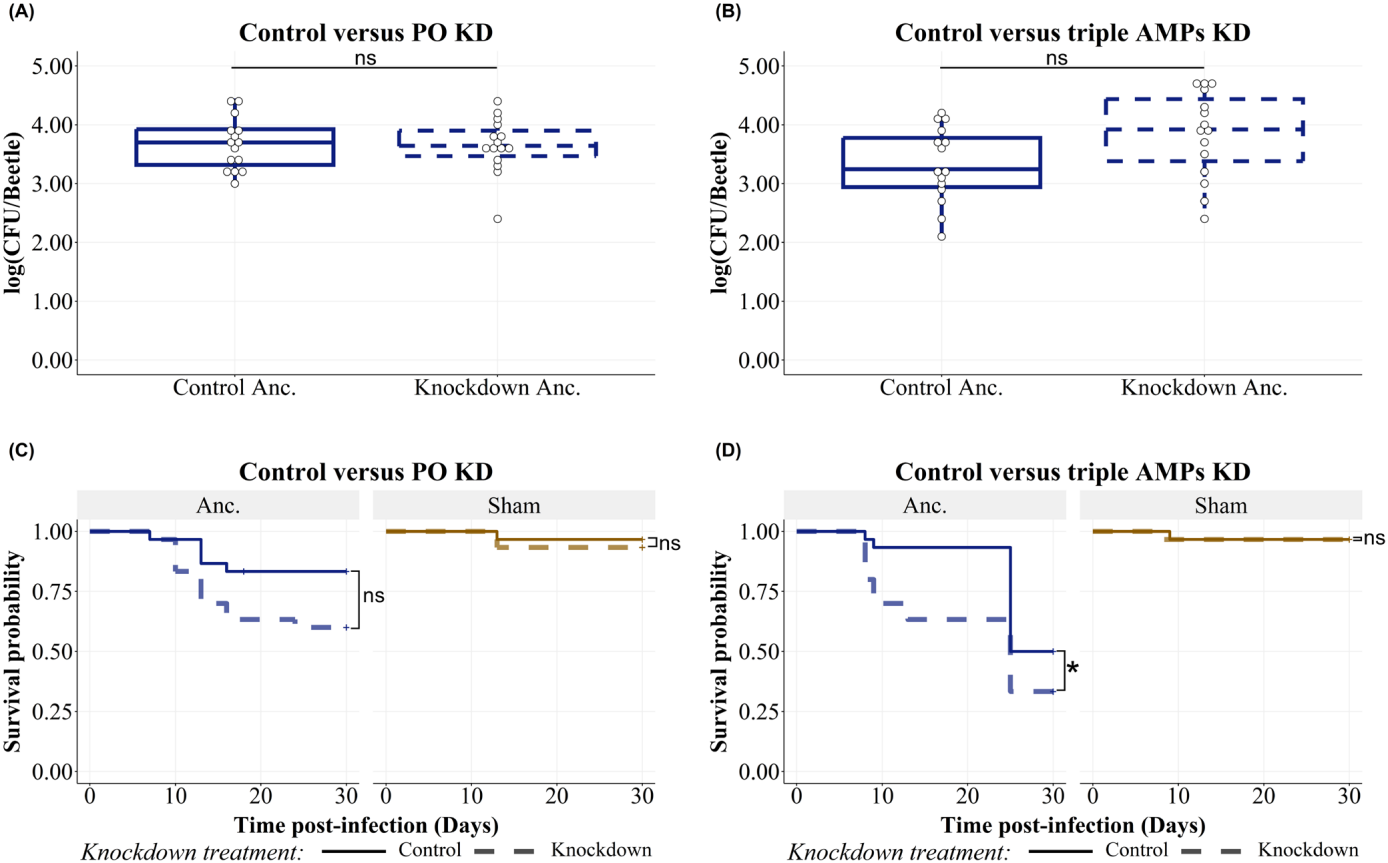
(A, B) Bacterial load of the ancestor *Staphylococcus aureus* strain (SH1000) in *Tenebrio molitor* recovered 7‐days post‐infection. The bacterial load recovered 1‐day post‐infection is represented in Figure S5. The colony‐forming units (CFUs) recovered from 100 μL of hemolymph are represented on a log scale by box plots showing quartiles and medians. The bars represent the 1.5 interquartile. Control ancestor (Anc). (solid outline) refers to the bacterial load of *S. aureus* in beetles that receive control RNAi. (A) The PO knockdown (dashed outline) did not affect in vivo colonization at any time point (linear model of box–cox transformed bacterial loads; prophenoloxidase knockdown treatment: *F*_1,57_ = 0.1351, *p* = 0.6823; time point: *F*_1,57_ = 31.9626, *p* < 0.0001). Knockdown Ancs (dashed outline) refers to the bacterial load of *S. aureus* in beetles with knockdown expression of prophenoloxidase. (B) The triple AMP knockdown (dashed) did not affect in vivo colonization at any time point compared to beetles with normal expression of tenecin 1, 2, and 4. Control (solid) (linear model of box–cox transformed bacterial load; T1T2T4 knockdown treatment: *F*_1,57_ = 4.7655, *p* = 0.033; time point: *F*_1,57_ = 12.02, *p* = 0.001)and knockdown Anc. (dashed outline) refer to the bacterial load of *S. aureus* in beetles with knockdown expression of tenecin 1, 2, and 4. (C, D) Survival of *T. molitor* infected with ancestor *Staphylococcus aureus* strain SH1000. The *p* value is calculated by the log rank test of differences of the censored survival curves computed by function “survdiff” from package “survival” in R. (C) The survival of beetles injected with the ancestor “Anc.” *S. aureus* (*X* ^2^ = 19.1, df = 3, *p* = 0.0003) tends to decrease by PO knockdown (*X* ^2^ = 19.1, df = 3, *p* = 0.09), while the sham‐infected (beetles injected with ringer solution) did not vary by knockdown treatment (*X* ^2^ = 19.1, df = 3, *p* = 0.56). Control (solid) and knockdown (dashed) refer to the beetles with normal or knockdown PO expression, respectively. (D) Survival of *T. molitor* infected with ancestor strain (*X* ^2^ = 46.6, df = 3, *p* < 0.0001), Anc., decreased by triple AMP knockdown (dashed) compared to beetles with normal expression of tenecin 1, 2, and 4 (*X* ^2^ = 46.6, df = 3, *p* = 0.04), control (solid), while the sham‐infected did not vary significantly by knockdown treatment (*X* ^2^ = 46.6, df = 3, *p* = 0.97). Control (solid) and knockdown (dashed) refer to the beetles with normal or knockdown tenecins (1, 2, and 4) expression, respectively (*n* = 15 beetles/group for A‐B and *n* = 30 beetles/group for C‐D divided into three replicates; ns: *p* > 0.05 and *: *p* < 0.05).

The in vivo survival of the ancestral strain of the pathogen *S. aureus* (SH1000) in the insect host ( *T. molitor* ) was neither affected by knockdown of the two phenoloxidase genes PO1 and PO2 (Figure 1A) nor by the combined knockdown treatment of tenecin 1 (defensin), tenecin 2 (coleoptericin), and tenecin 4 (attacin) genes (Figure 1B) when compared to individuals with normal expression levels 1 or 7‐days post‐infection (data for day 1 are presented for this (Figure S5) and all subsequent experiments in the supplement only (Figure S6)). Please note that the application of RNAi and hence also the controls differ between the PO and the AMP knockdowns (for more detail, see the Section 2). They can therefore not be directly compared.

The survival of the host insects, when infected with the same strain of *S. aureus* (Figure 1C), tended to decrease by 23% when PO was knocked down (*Χ* ^2^ = 19.1, df = 3, *p* = 0.09), with a mean age at death after injection of 12.9 days (survival rate 60%, number of death events = 12 of 30 beetles). Beetles that received control RNAi and peptidoglycan injections and infected with SH1000 showed a mean age at death after injection of 12.4 days (survival rate 83.33%, number of death events = 5 of 30 beetles). The survival of sham‐infected PO knockdown procedural control beetles (survival rate 93.3%, number of death events = 2 of 30 beetles) did not differ compared to those beetles that received the control dsRNA (survival rate 96.67%, number of death events = 1 of 30 beetles, *Χ* ^2^ = 19.1, df = 3, *p* = 0.56), both with mean age at death after injection of 13 days.

*Tenebrio molitor* with T1T2T4 knockdown infected with ancestral *S. aureus* strain had a mean age at death after injection of 16.3 days compared to beetles injected with control RNAi with a mean age at death after injection of 22.8 days (*Χ* ^2^ = 46.6, df = 3, *p* = 0.04, Figure 1D). The observed result is due to both bacterial infection and knockdown treatment because the survival of T1T2T4 knockdown sham‐infected beetles (procedural control) was not affected (*Χ* ^2^ = 46.6, df = 3, *p* = 0.60).

### 3.2 The Tenecin 1‐Resistant *Rpo* *Staphylococcus aureus* Mutant is Cross‐Sensitive to Phenoloxidase But Cross‐Resistant to the T1T2T4 Defense Cocktail

**FIGURE 2:**
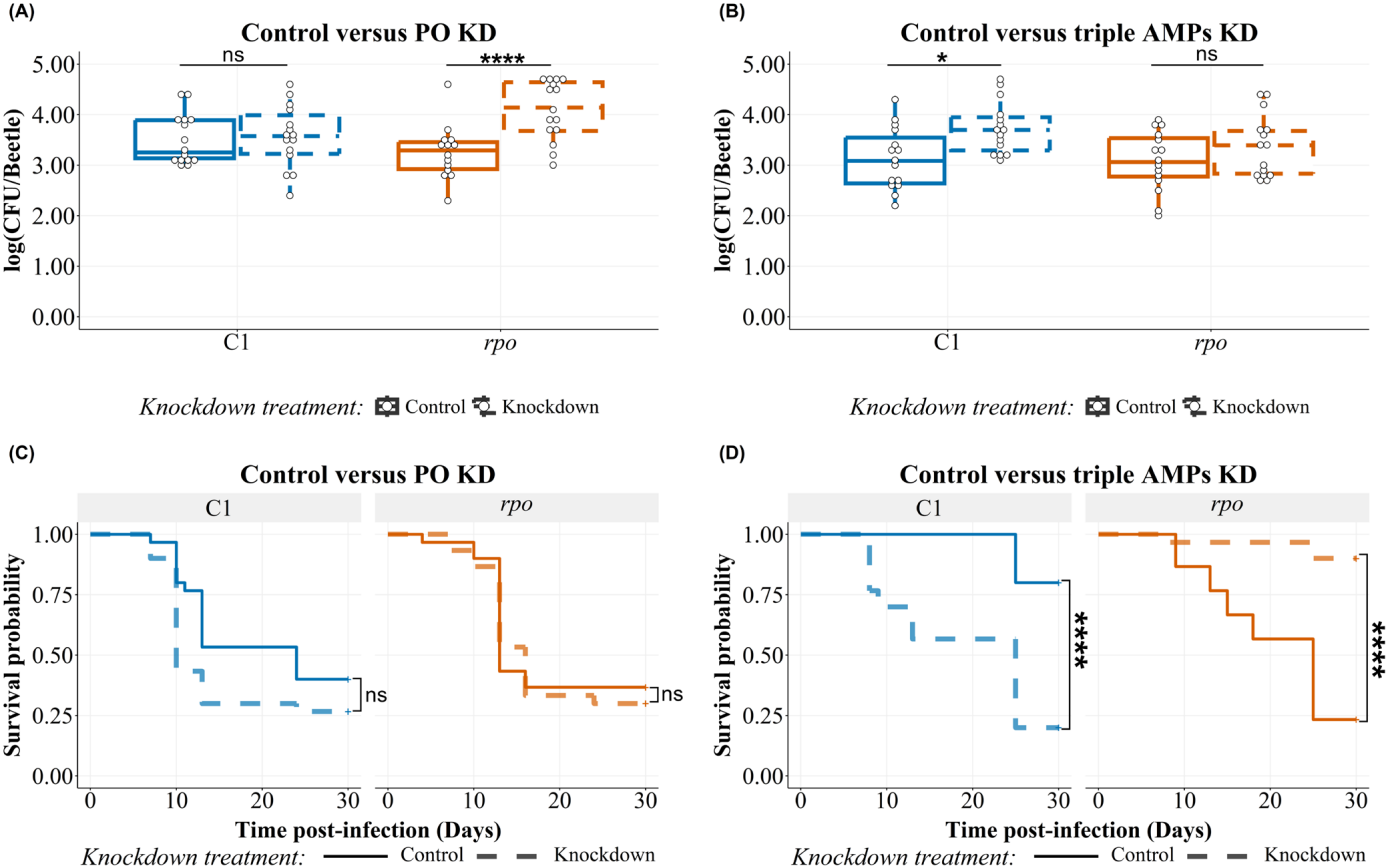
(A, B) Bacterial load of the tenecin 1 (defensin)‐resistant *Staphylococcus aureus* harboring a mutation in *rpo* operon in *Tenebrio molitor* 7‐days post‐infection. The bacterial load recovered 1‐day post‐infection is represented in Figure S6. The colony‐forming units (CFUs) recovered from 100 μL of hemolymph are represented on a log scale by box plots showing quartiles and medians. The bars represent the 1.5 interquartile. C1 refers to bacterial load of the tenecin‐sensitive control *S. aureus* (line 1, Table 1). *rpo* refers to the bacterial load of tenecin‐resistant *S. aureus* with mutation in *rpo* operon. Statistical pairwise comparison results are shown in Table 2. (A) The phenoloxidase knockdown PO KD (dashed outline) increased the colonization of *rpo* mutant (*p* = 0.0001), unlike the unselected control C1. The bacterial load is detected in either beetles with normal (control) or knockdown expression of prophenoloxidase (knockdown), represented by solid and dashed outlines, respectively. (B) The triple AMP knockdown (dashed) did not affect colonization of tenecin‐resistant *S. aureus* , *rpo*, by day‐7 post‐infection compared to beetles with normal expression of tenecin 1, 2, and 4, control (solid), unlike the unselected control C1, which increased significantly (*p* = 0.03). The bacterial load is detected in either beetles with normal (control) or knockdown expression of tenecin 1, tenecin 2, and tenecin 4 (knockdown). (C, D) Survival of *T. molitor* infected with *Staphylococcus aureus* strain harboring a mutation in *rpo* operon. The *p* value is calculated by the log rank test of differences of the censored survival curves computed by function “survdiff” from package “survival” in R. (C) Survival of *T. molitor* was affected neither by PO knockdown nor by bacterial AMP sensitivity (*X* ^2^ = 5.5, df = 3, *p* = 0.1). Control (solid) and knockdown (dashed) refer to the beetles with normal or knockdown PO expression, respectively. (D) Triple AMP knockdown treatment (*X* ^2^ = 53.5, df = 3, *p* < 0.0001) (dashed) increased the survival rate of *T. molitor* infected with *rpo* mutant compared to control (*p* < 0.0001), beetles with normal expression of tenecin 1, 2, and 4, in contrary to those infected with the unselected control tenecin‐sensitive strain, C1 (*p* < 0.0001). Control (solid) and knockdown (dashed) refer to the beetles with normal or knockdown tenecins (1, 2, and 4) expression, respectively (*n* = 15 beetles/group for A‐B and *n* = 30 beetles/group for C‐D divided into three replicates; ns: *p* > 0.05, *: *p* < 0.05 and ****: *p* ≤ 0.0001).

The bacterial load 1‐day post‐infection in host insects injected with *S. aureus* that belongs to line 1 (Table 1) including the control strain C1 and *rpo* mutants neither differ with PO knockdown treatment nor with strain sensitivity toward AMPS (Figure S6A, Table 2A). At 7‐days post‐infection, knocking down the phenoloxidase genes increased the bacterial load of bacteria harboring a mutation in the *rpo* operon compared to that of control beetles (Figure 2A, Table 2A).

**TABLE 2:** Statistical pairwise comparisons for CFUs recovered from infected beetles at different time points reported in Figures 1, 2, 3, 4 and Figures S6 and S7.

Contrast of bacterial load in host with different RNAi treatment	Bacterial strain	Time point	Estimated marginal means difference	SE	DF	T. ratio	Adjusted *p* (FDR)
A							
PO KD vs. Control	C1	Day1	0.799	1.23	446	0.649	0.8268
	C1	Day7	−0.388	1.23	446	−0.315	0.8601
	*rpo*	Day1	2.512	1.38	446	1.824	0.1572
	*rpo*	Day7	6.462	1.38	446	4.693	0.0001
	C2	Day1	0.483	1.07	446	0.454	0.8417
	C2	Day7	−0.916	1.07	446	−0.859	0.7813
	*pmt*	Day1	−0.674	1.65	446	−0.407	0.8417
	*pmt*	Day7	−4.714	1.65	446	−2.849	0.0184
	*pmt‐rpo*	Day1	−0.905	1.4	446	−0.649	0.8268
	*pmt‐rpo*	Day7	−4.041	1.4	446	−2.896	0.0184
	*nsa*	Day1	0.673	1.46	446	0.459	0.8417
	*nsa*	Day7	−3.85	1.46	446	−2.63	0.0283
	C3	Day1	−0.219	1.45	446	−0.151	0.9076
	C3	Day7	−3.194	1.45	446	−2.196	0.0764
	*nsa‐rpo*	Day1	−0.245	2.11	446	−0.116	0.9076
	*nsa‐rpo*	Day7	7.012	2.11	446	3.321	0.0078
B							
T1T2T4 KD vs. Control	C1	Day1	2.864	1.84	445	1.558	0.2385
	C1	Day7	−5.011	1.84	445	−2.726	0.0328
	*rpo*	Day1	2.015	1.78	445	1.129	0.346
	*rpo*	Day7	2.259	1.78	445	1.266	0.3124
	C2	Day1	2.985	1.9	445	1.574	0.2385
	C2	Day7	−4.78	1.9	445	−2.521	0.0385
	*pmt*	Day1	−2.916	2.35	445	−1.242	0.3124
	*pmt*	Day7	−6.187	2.31	445	−2.683	0.0328
	*pmt‐rpo*	Day1	2.927	1.95	445	1.501	0.2385
	*pmt‐rpo*	Day7	1.883	1.95	445	0.966	0.412
	*nsa*	Day1	0.458	1.5	445	0.305	0.7606
	*nsa*	Day7	−2.739	1.5	445	−1.822	0.1844
	C3	Day1	−1.059	1.87	445	−0.567	0.6529
	C3	Day7	−4.964	1.87	445	−2.656	0.0328
	*nsa‐rpo*	Day1	−0.758	1.85	445	−0.41	0.7278
	*nsa‐rpo*	Day7	−5.283	1.85	445	−2.854	0.0328

*Note:* The estimated marginal means of bacterial load difference between beetles with normal versus knockdown expression of (A) phenoloxidase and (B) the AMPs tenecin 1, tenecin 2, and tenecin 4 were computed from the contrasts between factors using “contrast” function from “lsmeans” package. The *p* values reported here are adjusted using the false discovery rate (FDR) method.

The bacterial load of hosts injected with the tenecin‐sensitive unselected control (C1) measured 7‐days post‐infection was significantly higher with T1T2T4 knockdown treatment compared to that of control beetles (Figure 2B, Table 2B). By contrast, in the case of insects infected with *rpo* mutant bacteria, bacterial load at 7 days did not change with the T1T2T4 knockdown treatment (Figure 2B, Table 2B). Neither the bacterial load of the tenecin‐sensitive C1 strain nor the tenecin 1‐resistant *rpo* mutants varied by AMP knockdown 1‐day post‐infection (Figure S6B, Table 2B). While we have different controls for different strains because of the selection history of these strains, we provide a comparison between strains in different host knockdown phenotypes by computing the effect sizes of bacterial loads for all strains used in our study in the supplement (see Figure S7).

The survival of *T. molitor* infected with *S. aureus* that belongs to line 1 was neither affected by bacterial genotype nor by phenoloxidase knockdown treatment (*Χ* ^2^ = 5.5, df = 3, Figure 2C). However, hosts infected with unselected control bacteria C1 followed the same survival pattern (Figure 2D) as those infected with the ancestral strain (Figure 1D). The T1T2T4 knockdown treatment increased the mortality of beetles infected with C1 *S. aureus* by 60% with a mean age at death after the injection of 16.7 days compared to the beetles with normal expression levels of T1T2T4 with mean age at death after the injection of 25 days (*Χ* ^2^ = 53.5, df = 3, *p* < 0.0001). *T. molitor* infected with the *rpo* mutant had a higher mortality rate in hosts with normal expression levels of T1T2T4 with a mean age at death after injection of 19.3 days. However, beetles with the knockdown expression of T1T2T4 and infected with *rpo* mutant *S. aureus* showed a significantly decreased mortality rate by 66.67% with a mean age at death after the injection of 18.4 days (*Χ* ^2^ = 53.5, df = 3, *p* < 0.0001).

### 3.3 AMP‐Resistant *Staphylococcus aureus* Harboring a Mutation in *Pmt, Pmt‐Rpo,* or *Nsa* Operons Shows Cross‐Sensitivity Toward Phenoloxidase While Only *Pmt* Mutants Are Cross‐Sensitive to One or More Components of the Defense Cocktail T1T2T4

**FIGURE 3:**
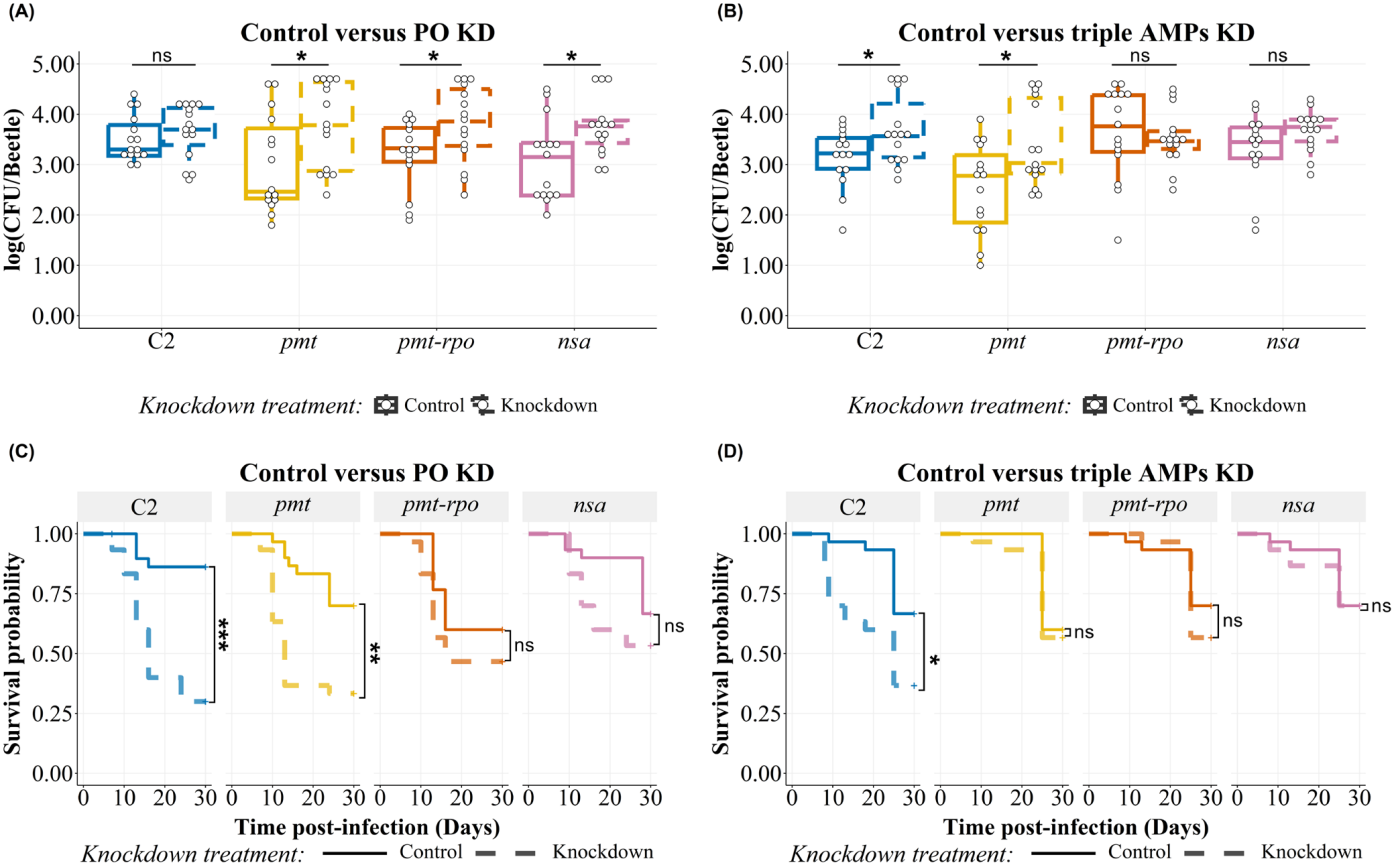
(A, B) Bacterial load of tenecin‐resistant *Staphylococcus aureus* (line 2, Table 1) 7‐days post‐infection harboring a mutation in *pmt, pmt‐rpo*, or *nsa*operon compared to the tenecin‐sensitive *S. aureus* C2. The bacterial load recovered 1‐day post‐infection is represented in Figure S6. The colony‐forming units (CFUs) recovered from 100 μL of hemolymph are represented on a log scale by box plots showing quartiles and medians. The bars represent the 1.5 interquartile. Statistical pairwise comparison results are shown in Table 2. (A) The PO knockdown (dashed outline) increased colonization of tenecin‐resistant *S. aureus* in beetles by day 7 for all mutants compared to those with normal expression levels of prophenoloxidase, control (solid outline), unlike the unselected control tenecin‐sensitive *S. aureus* strain C2. Control (solid) and knockdown (dashed) refer to the beetles with normal or knockdown PO expression, respectively. (B) The triple AMP knockdown (dashed outline) increased the colonization of AMP‐resistant *S. aureus* by day‐7 post‐infection for the *pmt* mutant and the unselected control C2 compared to beetles with normal expression of tenecin 1, 2, and 4, control (solid outline). The bacterial load is detected in either beetles with normal (control) or knockdown expression of tenecin 1, tenecin 2, and tenecin 4 (knockdown). (C, D) The survival rate of beetles infected with tenecin‐resistant strains harboring mutation in *pmt*, *pmt‐rpo*, or *nsa* operon compared to the tenecin‐sensitive *S. aureus* C2. The *p* value is calculated by the log rank test of differences of survival. (C) PO KD treatment, knockdown (dashed line), decreased the survival rate of *T. molitor* infected with *pmt* mutant (*p* = 0.0047) as well as those infected with the unselected control strain, C2 (*p* = 0.0002) compared to those with normal expression levels of prophenoloxidase, control (solid line). The mortality rates of *T. molitor* infected with *pmt‐rpo* and *nsa* mutants were not affected by PO knockdown (*X* ^2^ = 37.5, df = 7, *p* < 0.0001). Control (solid) and knockdown (dashed) refer to the beetles with normal or knockdown PO expression, respectively. (D) The triple AMP knockdown treatment (dashed) decreased the survival rate of *T. molitor* infected with unselected control strain, C2 (*p* = 0.04) compared to beetles with normal expression of tenecin 1, 2, and 4, control (solid). The mortality rates of *T. molitor* infected with *pmt*, *pmt‐rpo*, and *nsa* mutants were not affected by the triple AMP knockdown (*X* ^2^ = 20.4, df = 7, *p* = 0.005). Control (solid) and knockdown (dashed) refer to the beetles with normal or knockdown tenecins (1, 2 and 4) expression, respectively (*n* = 15 beetles/group for A‐B and *n* = 30 beetles/group for C‐D divided into three replicates; ns: *p* > 0.05, *: *p* < 0.05, **: *p* ≤ 0.01 and ***: *p* ≤ 0.001).

The knockdown of phenoloxidase did not affect the in‐host survival of any of the *pmt*, *pmt‐rpo*, or *nsa* mutants nor the unselected control 1‐day post‐infection (Figure S6C, Table 2A). However, 7‐days post‐infection, the bacterial load of tenecin 1‐resistant *pmt* and *pmt‐rpo* and tenecin 1‐ and tenecin 2‐resistant *nsa* mutant *S. aureus* stains increased significantly with PO knockdown treatment (Figure 3A, Table 2A).

Similarly, the number of recovered CFUs of the tenecin 1‐resistant *S. aureus* with a mutation in the *pmt* operon increased significantly with the T1T2T4 knockdown treatment 7‐days post‐infection (Figure 3B, Table 2B). However, at the same time point post infection, the infection load of the tenecin 1‐resistant *S. aureus* harboring an additional mutation in the *rpo* operon (*pmt‐rpo*) did not change with the T1T2T4 knockdown. Therefore, the *pmt* mutant is sensitive, while the *pmt‐rpo* mutant is in‐sensitive to coleoptericin (tenecin 2), attacin (tenecin 4) or both. The tenecin 1‐ and tenecin 2‐resistant *S. aureus* harboring a mutation in *nsa* operon showed a similar pattern as *pmt‐rpo* mutant as bacterial load was not affected by the T1T2T4 knockdown treatment (Table 2B).

Knockdown of phenoloxidase affected the survival of *T. molitor* infected with *S. aureus* (Figure 3C) that belongs to line 2 (Table 1). The mortality of *T. molitor* infected with *S. aureus* with a mutation in *pmt* operon increased significantly with PO knockdown treatment (*Χ* ^2^ = 37.5, df = 7, *p* = 0.004). Beetles with a knockdown expression of phenoloxidase and infected with tenecin‐sensitive control *S. aureus* (C2) showed reduced survival compared to control beetles (*Χ* ^2^ = 37.5, df = 7, *p* = 0.0002). This was not found for *pmt‐rpo* (*Χ* ^2^ = 37.5, df = 7, *p* = 0.223) and *nsa* (*Χ* ^2^ = 37.5, df = 7, *p* = 0.223) mutants. Therefore, phenoloxidase limits the virulence of *pmt* mutant and unselected control (C2).

The survival of beetles infected with unselected control bacteria (C2) was significantly lower with host T1T2T4 knockdown treatment (Figure 3D) compared to beetles injected with control RNAi (*Χ* ^2^ = 20.4, df = 7, *p* = 0.04). However, the T1T2T4 knockdown treatment did not affect the mortality rate of the line 2 descended T1‐ and T1T2‐selected *S. aureus* strains, which (T1‐2L, T1‐2S, and T1T2‐2) harbor mutations in *pmt* (*Χ* ^2^ = 20.4, df = 7, *p* = 0.95), *pmt‐rpo* (*Χ* ^2^ = 20.4, df = 7, *p* = 0.90), and *nsa* (*Χ* ^2^ = 20.4, df = 7, *p* = 0.95), respectively.

### 3.4 The Tenecin 1‐ and Tenecin 2‐Resistant *Nsa‐Rpo* *Staphylococcus aureus* Mutant Are Cross‐Resistant to Phenoloxidase, While Cross‐Sensitive to the In Vivo Defense Cocktail T1T2T4

In‐host survival 1‐day post‐infection of the T1T2‐sensitive unselected control *S. aureus* (C3) was not affected by the knockdown of host phenoloxidase at any time point post infection (Figure S6E, Table 2A).

**FIGURE 4:**
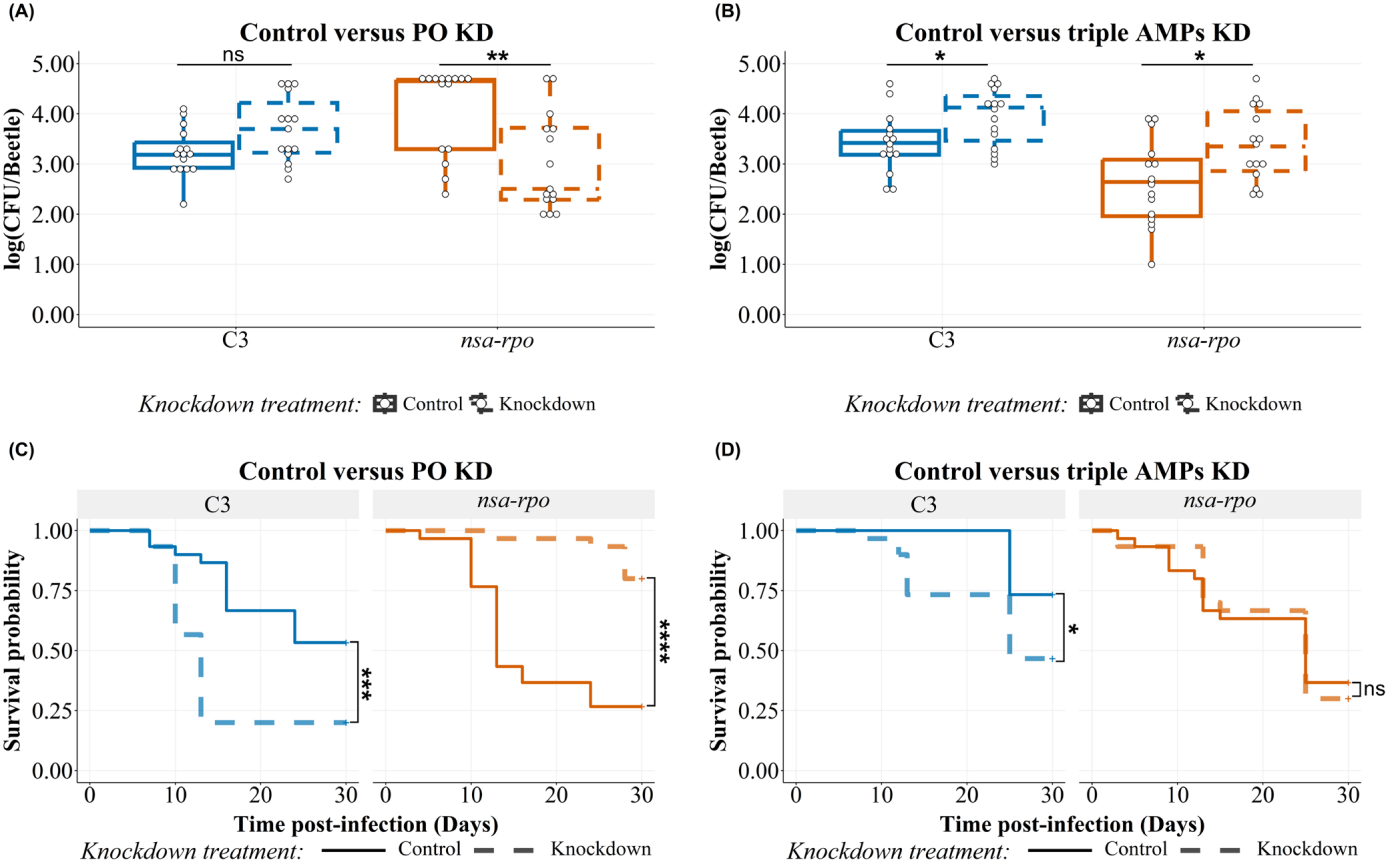
(A, B) Bacterial load of tenecin‐resistant *Staphylococcus aureus* (line 3, Table 1) harboring a mutation in *nsa‐rpo* operon compared to the tenecin‐sensitive *S. aureus* C3, 7‐days post‐infection. The bacterial load recovered 1‐day post‐infection is represented in Figure S6. The colony‐forming units (CFUs) recovered from 100 μL of hemolymph are represented on a log scale by box plots showing quartiles and medians. The bars represent the 1.5 interquartile. Statistical pairwise comparison results are shown in Table 2. (A) The PO knockdown (dashed) decreased the colonization of tenecin‐resistant *nsa‐rpo S. aureus* mutants by day‐7 post‐infection (*p* = 0.0078) compared to those with normal expression levels of prophenoloxidase (solid). The tenecin‐sensitive C3 *S. aureus* strain was not affected by PO knockdown treatment (*p* = 0.0764). Control (solid) and knockdown (dashed) refer to the beetles with normal or knockdown PO expression, respectively. (B) The triple AMP knockdown (dashed) increased the colonization of both tenecin‐resistant *S. aureus nsa‐rpo* mutants (*p* = 0.0328) and the tenecin‐sensitive unselected control strain C3 by day 7 (*p* = 0.0328) compared to beetles with normal expression of tenecin 1, 2, and 4, control (solid). The bacterial load is detected in either beetles with normal (control) or knockdown expression of tenecin 1, tenecin 2, and tenecin 4 (knockdown). (C, D) The survival rate of beetles infected with tenecin‐resistant strains harboring mutation in *nsa‐rpo* mutant operon compared to the tenecin‐sensitive *S. aureus* C3. The *p* value is calculated by the log rank test of differences of survival. (C) PO treatment (dashed) increased the survival rate of *T. molitor* infected with *nsa‐rpo* mutant (*p* < 0.00001), while decreased the survival rate of those infected with the unselected control strain, C3 (*p* = 0.0007) compared to those with normal expression levels of prophenoloxidase, control (solid) (*X* ^2^ = 37, df = 3, *p* < 0.0001). Control (solid) and knockdown (dashed) refer to the beetles with normal or knockdown PO expression, respectively. (D) Triple AMP knockdown treatment (dashed) decreased the survival rate of *T. molitor* infected with the unselected control strain, C3 (*p* = 0.02) compared to beetles with normal expression of tenecin 1, 2, and 4, control (solid). However, it did not affect those infected with *nsa‐rpo* mutant (*X* ^2^ = 14.8, df = 3, *p* = 0.002). Control (solid) and knockdown (dashed) refer to the beetles with normal or knocked‐down tenecins (1, 2 and 4) expression, respectively (*n* = 15 beetles/group for A‐B and *n* = 30 beetles/group for C‐D divided into three replicates; ns: *p* > 0.05, *: *p* < 0.05, ***: *p* ≤ 0.001 and ****: *p* ≤ 0.0001).

At normal levels of PO expression, the bacterial load at 7‐days post‐infection of hosts injected with T1T2‐selected *S. aureus* (T1T2‐3) harboring mutations in both *nsa* and *rpo* operons was significantly higher than when host PO was knocked down (Figure 4A, Table 2A). The knockdown had no effect at earlier infection time point.

When host T1T2T4 had been knocked down, the bacterial load of the unselected control (C3) or *S. aureus* (T1T2‐3) harboring a mutation in both *nsa* and *rpo* operons was significantly higher compared to control beetles 7‐days post‐infection (Figure 4B, Table 2B). However, 1‐day post‐infection, the bacterial load of both strains (C3 and *nsa‐rpo*) was not affected by the knockdown treatment of T1T2T4 genes (Figure S6F, Table 2B).

In *T. molitor* infected with *S. aureus* descended from line‐3 (listed in Table 1), host survival was affected by knocking down the expression of phenoloxidase gene regardless of the strain sensitivity to tenecins (Figure 4C). The mortality of hosts injected with T1T2‐sensitive unselected control (C3) increased approximately 33% with PO knockdown treatment (*Χ* ^2^ = 37, df = 3, *p* = 0.001). However, the mortality of T1T2‐resistant *nsa‐rpo* mutant injected insects decreased 53.3% with PO knockdown treatment (*Χ* ^2^ = 37, df = 3, *p* < 0.0001).

Similar to the ancestor SH1000 and the unselected control strains C1 and C2, the survival of beetles infected with the unselected control strain C3 (Table 1) decreased significantly with T1T2T4 knockdown treatment (Figure 4D) compared to control beetles (*Χ* ^2^ = 14.8, df = 3, *p* = 0.02). The host mortality rate caused by the *nsa‐rpo* mutant infection was high regardless of knockdown treatment (*Χ* ^2^ = 14.8, df = 3, *p* = 0.91), with mean age at death after injection of 16.5 and 18.4 days for the beetles with normal and knockdown expression levels of T1T2T4, respectively.

## 4 Discussion

To understand the in vivo pathogenesis of AMP‐resistant bacteria, we used *Tenebrio molitor* as a host and found that the majority of AMP‐resistant *S. aureus* showed collaterally increased sensitivity to other immune effectors in vivo. All our strains with AMP resistance mutations, except for the strain with a double mutation in both the *nsa* and *rpo* operons, were sensitive to phenoloxidase. The tenecin 1‐ and tenecin 2‐resistant *nsa‐rpo* double mutant *S. aureus* , however, showed sensitivity to other AMPs in vivo. The latter result was also found for strains with a mutation in the *pmt* operon that were sensitive to one or more components of the in vivo AMP defense cocktail, as revealed by simplifying this cocktail. In short, all AMP‐resistant mutants investigated here showed collaterally increased sensitivity in vivo to at least one of the other components of the insect immune system that we experimentally manipulated using RNAi. Taken together, our results are consistent with the idea that bacterial resistance to host immune effectors can be costly: resistance to one immune effector shows negative pleiotropy with one or more other immune effectors. This contrasts with some findings on antimicrobial resistance, where resistant strains can show increased in vivo fitness and virulence (Roux et al. 2015).

We found that for all the *S. aureus* strains selected for AMP resistance studied, except for those harboring mutations in the *nsa*‐*rpo* operons, knockdown of phenoloxidase increased the bacterial load. This shows the important role of phenoloxidase in insect antibacterial defenses. The effectiveness of phenoloxidase in insect immune responses is consistent with the findings of Ayres and Schneider (2008) who reported that disrupting the melanization pathways in *D. melanogaster* (CG3066) increased both the bacterial load and mortality in the case of *Salmonella typhimurium* and *Listeria monocytogenes* infections. However, phenoloxidase is not always effective in this way; for example, phenoloxidase knockdown did not influence the bacterial load of *Pseudomonas rettgeri* (Duneau et al. 2017) nor of *E. coli* (Ayres and Schneider 2008) in *D. melanogaster* . Here, we report that also different strains of the same pathogen display different sensitivity to the melanization pathway, almost certainly caused by the selected mutations providing AMP resistance. The phenoloxidase response is activated via the recognition of membrane structural components of bacterial cells (Cerenius, Lee, and Söderhäll 2008). AMP resistance usually includes modifications of the bacterial membranes (Andersson, Hughes, and Kubicek‐Sutherland 2016). Therefore, it is possible that such cell surface modifications render the AMP‐resistant *S. aureus* more susceptible to recognition by the PPO‐activating system resulting in increased sensitivity to phenoloxidase‐dependent immune responses. It is worth noting that the T1T2‐resistant *nsa‐rpo* mutants, the only mutants in our set that are sensitive to phagocytosis (El Shazely et al. 2019), also show resistance to phenoloxidase. Moreover, knockdown of the beetle phenoloxidase prior to infection with *nsa‐rpo* mutants decreased both bacterial load and virulence in the host. A possible explanation for the reduction in virulence of the *nsa‐rpo* mutants that we observed is reduced collateral damage to the Malpighian tubules (an equivalent of the human kidney) that can be caused by phenoloxidase (Khan, Prakash, and Agashe 2017). To the best of our knowledge, no previous studies discussed the sensitivity of pathogens selected to endogenous AMPs toward phenoloxidase‐dependent immune responses.

Consistent with previous studies (Keshavarz, Zanchi, and Rolff 2023; Zanchi, Johnston, and Rolff 2017), we find a lot of variation in bacterial loads that is increasing over time in both control and knockdown beetles for the majority of bacterial strains tested. Similar increases in variation have even been found in *Drosophila* with isogenic backgrounds (Duneau et al. 2017) and *Drosophila* populations infected with one of either *Lactobacillus lactis* , *Providencia burholderia*, or *Enterobacter cloacae* (Franz et al. 2023; Hidalgo et al. 2022). While such patterns seem to be common, the underlying reasons for this are still unclear (Duneau and Ferdy 2022). Theoretical work suggests feedbacks between host and pathogens (Ellner et al. 2021), which awaits experimental testing. The reason for the differences in survival caused by control lines across experiments is more likely explained by the fact that not all experiments could be carried out simultaneously and that it cannot be ruled out that mutations occur during passaging. The most meaningful comparisons are therefore the comparisons within the experiments; this is reflected in our statistical models.

Another interesting observation in our data is that the differences in bacterial loads and their changes caused by knockdowns of either AMPs or phenoloxidase are not always mirrored in differences in survival (see Figure 3). It is unclear at the moment whether this is caused by a possible lack of statistical power for the *nsaS/R* mutants, for example. An alternative interpretation is that host resistance or tolerance, the ability of a host to curtail the damage of a given pathogen load (Kutzer and Armitage 2016), toward a pathogen can be a strain‐specific response, in our case related to a specific AMP resistance mutation. While this is intriguing, it clearly requires additional work.

The number of bacterial AMP resistance mechanisms seems limited and rather generic (Joo, Fu, and Otto 2016). Consequently, we expect that bacteria with evolved resistance to one AMP will also display cross‐resistance to other AMPs, especially in the case of AMPs with related structure. While not studied yet, it would be very interesting to study if and how such cross‐resistance contributes to the evolution of small but significant changes in AMP efficacy as reported in fruit flies (Unckless, Howick, and Lazzaro 2016). We previously reported that our tenecin‐resistant *S. aureus* showed cross‐resistance toward melittin and colistin but had increased sensitivity toward pexiganan and vancomycin (El Shazely et al. 2020; Makarova et al. 2018). In the present work, tenecin 1‐resistant *S. aureus* harboring a mutation in the *pmt* operon showed increased sensitivity to tenecins 2 and 4. The same result was found for the tenecin 1‐ and tenecin 2‐resistant *nsa‐rpo* *S. aureus* mutant strains. This finding explains the lower bacterial load of those particular two strains in vivo compared to other AMP‐resistant mutants at day‐14 post‐infection (El Shazely et al. 2019). Here, we showed that AMP resistance can confer either cross‐resistance or collateral sensitivity to the host's other AMPs, and again this was dependent on the resistance mutation.

The T1T2T4 knockdown did not influence the virulence of the AMP‐resistant strains except for the tenecin 1‐resistant *S. aureus* harboring a mutation in the *rpo* operon, where virulence was decreased compared to that of the unselected tenecin‐sensitive control. It is unclear why the *rpo* mutant caused a lower host mortality rate in the case of T1T2T4 knockdown treatment.

Given the notion that a single mutation can cause various phenotypic changes that regulate the evolution of resistance and susceptibility to various drugs simultaneously (Lázár et al. 2013), adaptations to one immune effector (e.g., AMPs) may result in increased sensitivity to another modulator defining the outcomes of the host–pathogen interaction. This might explain why AMP‐resistant bacteria are not abundant in nature (Spohn et al. 2019), although there is ample opportunity for resistance selection, for example, in environments such as the animal gut (Bevins and Salzman 2011). During the last decade, collateral sensitivity for bacterial strains under selection by pairs of antibiotics has been re‐described (Imamovic and Sommer 2013; Lázár et al. 2013). However, our study is the first to investigate the collateral sensitivity of bacteria that have evolved resistance to selected AMPs of the host (Bell and Gouyon 2003) to other host immune effectors. Our results suggest that overcoming the different hurdles to establish a successful infection (Hall, Bento, and Ebert 2017) can be hampered by antagonistic pleiotropy: resistance to one defense comes at the cost of increased sensitivity to another defense.

## Conflicts of Interest

The authors declare no conflicts of interest.

## Supporting information


Appendix S1:

## Data Availability

The raw data for all figures and the qPCR data for the RNAi knockdowns are available on refubium (https://refubium.fu‐berlin.de/?locale‐attribute=en), http://doi.org/10.17169/refubium‐38491.
