# Supplementary material for: A Trade‐Off Between Antimicrobial Peptide Resistance and Sensitivity to Host Immune Effectors in Staphylococcus aureus In Vivo
Source: Evol Appl. 2025 Feb 6;18(2):e70068. doi: 10.1111/eva.70068 (PMC11802329; doi:10.1111/eva.70068)
Supplement: Supplementary file 1 — Appendix S1: [file EVA-18-e70068-s001.zip › Supplementary information.docx]

**Supplementary information**

**A Trade-Off Between Antimicrobial Peptide Resistance and Sensitivity to Host Immune Effectors in *Staphylococcus aureus* In Vivo**

- 1. **Review of literature of mutations driving AMP resistance in tenecin-selected *S. aureus***

**Table S1.** Mutations previously reported (Makarova et al., 2018) in tenecin-selected *Staphylococcous aureus*. The tenecin-selected strains used in this study harbor a mutation in either *pmt* or *nsa* operons which in some strains is accompanied by a mutation in the *rpo* operon.

| Gene name | Function | Mutation | Tenecin-selected *S. aureus* strains used in the current study harboring a mutation in this operon |
| --- | --- | --- | --- |
| *pmt* | The ABC transporter, Pmt, exports bacterial toxins known as Phenol-soluble modulins (PSM) and is encoded by the *pmt* operon (Peschel & Otto, 2013).  The repressor PmtR negatively regulates the *pmt* expression through binding to the *pmt* promotor (Joo, Fu, & Otto, 2016).  Bacterial exposure to cationic AMPs induces the *pmt* expression (Li et al., 2007).  Pmt transporters prevent bacterial killing by human AMPs (Cheung et al., 2018). | Mutants with *pmt*R deletion are capable of active export of PSMs due to constitutive upregulation of pmtA-D (Joo et al., 2016).  Tenecin 1-resistance is likely mediated by *pmt*R mutations (Makarova et al., 2018).  Strains with mutations in *pmt*R showed high nisin A-resistance and some showed increased pathogenicity (Kawada-Matsuo, Le, & Komatsuzawa, 2021). | T1-2L (tenecin 1-resistant *S. aureus* with large morphology which harbors a mutation in the *pmt* operon)  T1-2S (tenecin 1-resistant *S. aureus* with small bacterial cells and have mutations in both the *pmt* and *rpo* operons) |
| *nsa* | The nisin susceptibility-associated two-component system NsaSR (also known as BceSR and BraSR), which controls two ABC transporters, BraDE and vraDE (Hiron, Falord, Valle, Débarbouillé, & Msadek, 2011; Yoshida et al., 2011), decreases susceptibility to several lantibiotics (Kawada-Matsuo et al., 2013). | *Staphylococcus aureus* with a single mutation in *nsa*RS have high resistance against nisin A (Arii, Kawada-Matsuo, Oogai, Noguchi, & Komatsuzawa, 2019).  Some of the *S. aureus* which showed resistance to tenecin 1 harbor mutations in the *nsa* operon (Makarova et al., 2018). | T1T2-2 (tenecin 1 plus tenecin 2-resistant *S. aureus* with mutations in *nsa* operon)  T1T2-3 (tenecin 1 plus tenecin 2-resistant *S. aureus* with mutations in *nsa* and *rpo* operons) |
| *rpo* | The *rpo* genes are known to encode for bacterial RNA polymerase β and β’ subunits (Lee, Nam, & Helmann, 2013; Zalenskaya et al., 1990). | Mutations in *rpo*B and *rpo*C have been reported in daptomycin and vancomycin resistance in *Staphylococcous aureus*. These strains have thickened cell walls and increased positive charge (Cui et al., 2010; Gómez Casanova, Siller Ruiz, & Muñoz Bellido, 2017).  Mutations in *rpo* genes are thought to adjust the bacterial global transcriptional profile rather than directly contributing to antimicrobial resistance (Ma et al., 2018). | T1-1L (tenecin 1-resistant *S. aureus* with large cell size and harbours mutation in *rpo* operon)  T1-2S (tenecin 1-resistant *S. aureus* with small cell size and harbours mutations in *pmt* and *rpo* operons)  T1T2-3 (tenecin 1 plus tenecin 2-resistant *S. aureus* with mutations in *nsa* and *rpo* operons) |

- 1. **Supplementary information to materials and methods**
  2. **Rearing of *Tenebrio molitor***

The mealworm beetles, *Tenebrio molitor,* were reared as previously described (El Shazely, Urbanski, Johnston, & Rolff, 2019). Briefly, *T. molitor* larvae were purchased from a commercial supplier who maintains big, outbred populations, and then kept at a density of 500 individuals per container at 25 °C in the dark. Cultures were kept with *ad libitum* access to wheat bran supplemented with fresh peeled apple pieces 2 to 3 times per week as a source of moisture. The rearing boxes were checked for pupae which were examined and sexed under a binocular dissecting microscope. Newly emerged female adults were maintained individually in grid boxes. We provided each with bran, piece of filter paper, and 1 mm^3^ piece of apple. Experiments were performed on 7 to 9 days old females with a weight ranging between 0.120 g and 0.190 g.

- 1. **Experimental design**


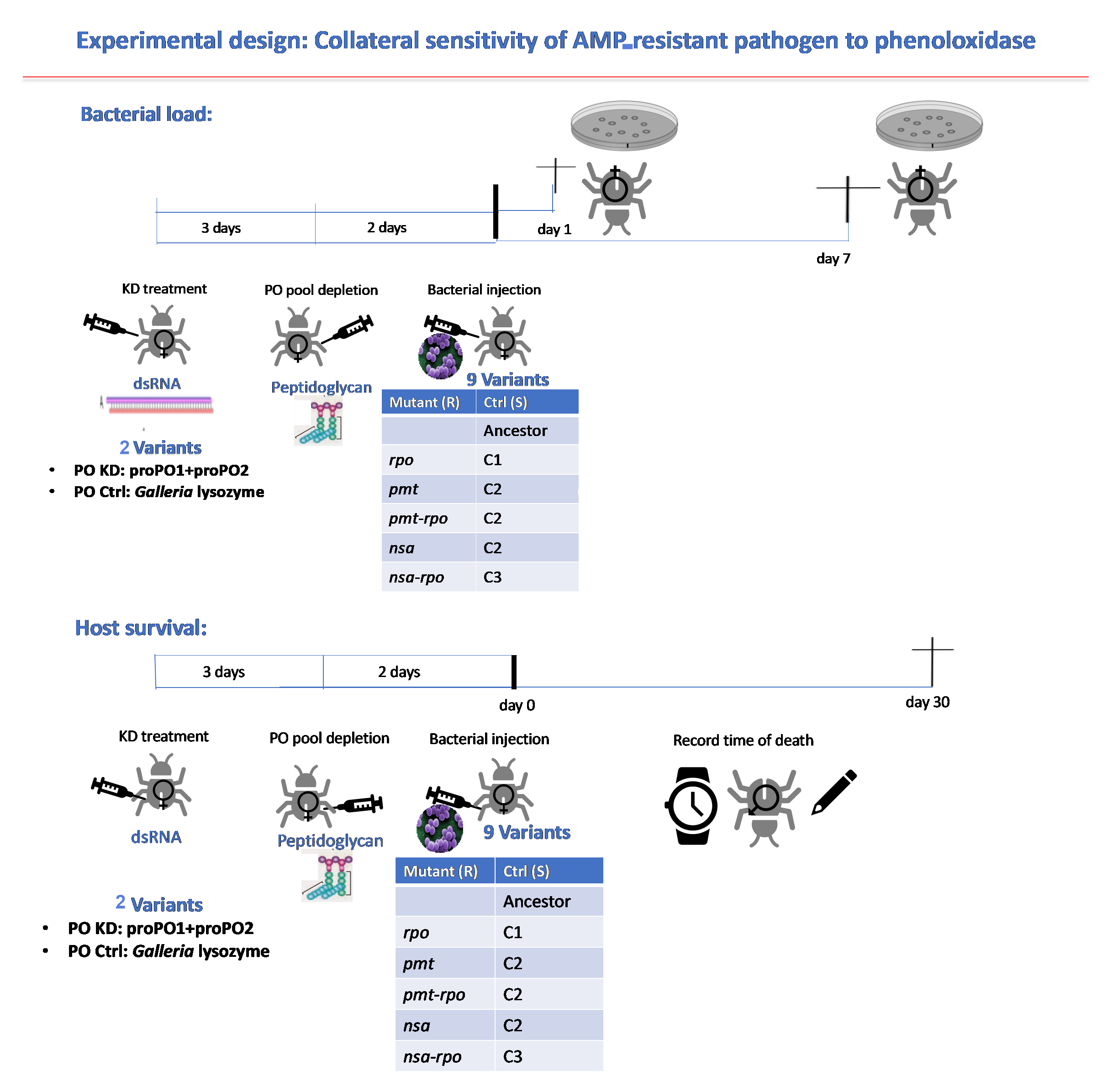


**Figure S1:** Collateral sensitivity of tenecin-resistant pathogen (*S. aureus*) toward phenoloxidase (one of the host’s pro-inflammatory responses). We aimed to investigate whether the evolution of AMP resistance in *S. aureus* results in increased or decreased bacterial survival inside the host in the presence or absence of phenoloxidase, which we experimentally altered using RNA interference (RNAi) of prophenoloxidase (PPO). We also monitored the host’s survival.


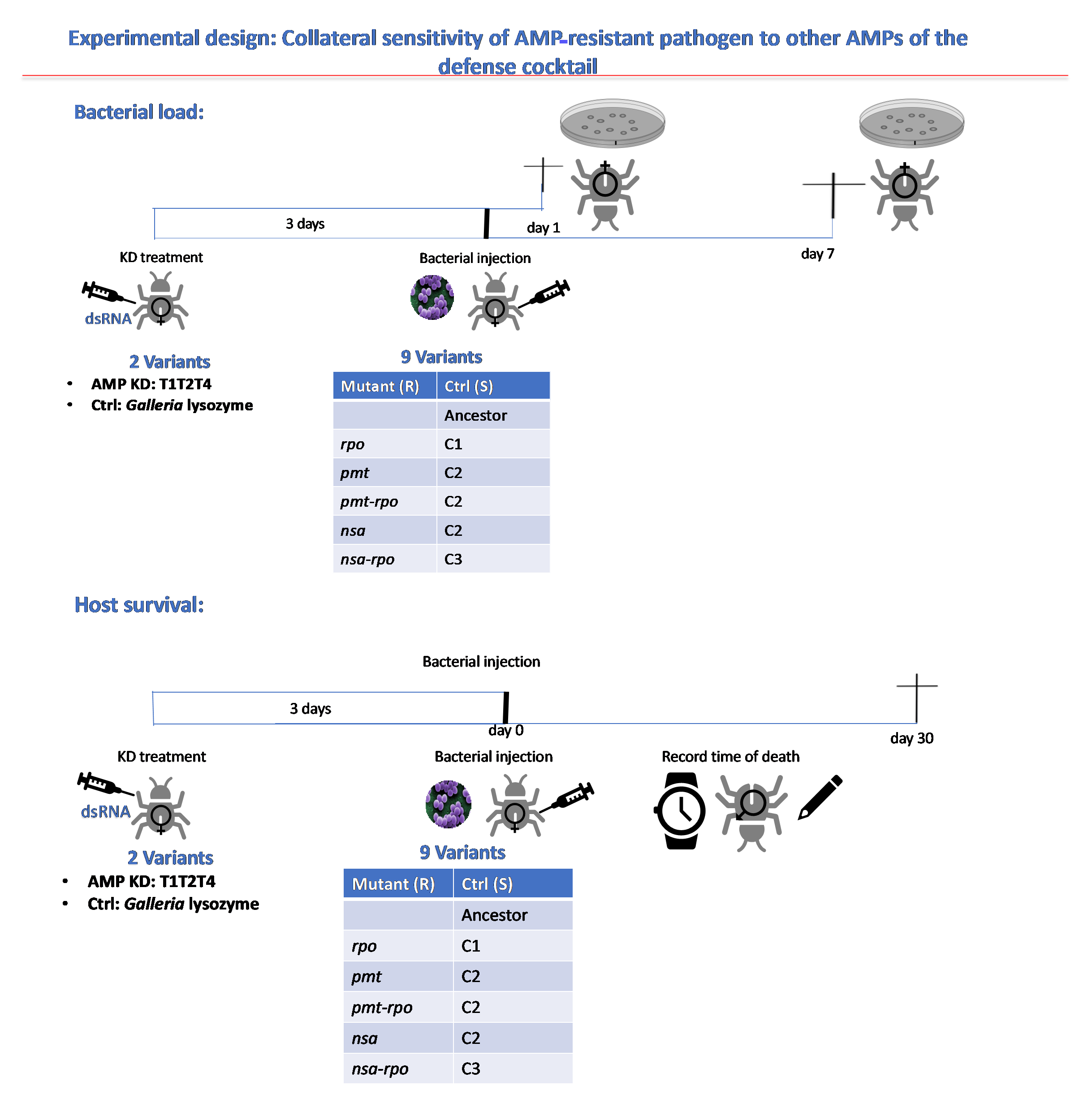


**Figure S2:** Collateral sensitivity of T1- and T1T2-resistant *S. aureus* toward T2 and/or T4. We aimed to investigate whether the evolution of resistance toward one- or two-AMPs changes sensitivity of the pathogen toward other AMPs. We experimentally altered the degree of AMP-based immune response using RNA interference (RNAi) of tenecin 1, tenecin 2 and tenecin 4 expression and quantified survival of both pathogen and host.

- 1. **Checking knockdown efficiency**
     1. **RNA extraction**

A quantitative real-time polymerase chain reaction (qRTPCR) was performed to test for the effectiveness of target genes knockdown on the mRNA extracted from the beetles’ fat bodies (an equivalent to the human liver).

Five random corpses of frozen beetles per treatment, stored at -80°C, were selected to check for dsRNA-based knockdown effectiveness. Over dry ice, 800-1000 µls of RNALater -ICE Frozen Transition Solution (Ambion life technologies, Cat. # AM7030, 4427575) were added to the frozen samples and stored in -20 °C for 24 h. Frozen corpses were then dissected under a dissecting microscope using sterile, RNAse-free tools. The fat bodies were isolated and stored in a 2 ml tube containing 1 ml TRIzol Reagent (Sigma-Aldrich, Cat # T9424-200ML). To grind the tissues, two sterile PCR-clean 3mm beads (Qiagen, Cat # 69997) were added to the TRIzol-fat bodies mixture. In a Retsch MM 400 homogenizer, the samples were run twice for 3 min at 30 Hz.

The homogenized samples were then incubated at room temperature for 5 min, then 400 µl Chloroform (Sigma-Aldrich, Cat # 472476) were added. The samples were vortexed vigorously and incubated at room temperature for 10 min. The samples were centrifuged at 14 000 g for 15 min. The upper aqueous phase was transferred into a new tube. Then, 1000 µl isopropanol (Carl Roth, Cat # 6752.4) were added and stored at -20°C overnight to precipitate nucleic acids. The samples were centrifuged at 20 000 g at 4 °C for 20 min. The supernatant was removed, and the pellet was washed by adding 1000 µl 75% ethanol (prepared from Carl Roth, Cat # 9065.3). The samples were centrifuged at 7 500 g for 5 min. For each sample, the supernatant was discarded, and the pellet was shortly air-dried. The palette was then solubilized in 88 µl nuclease-free water (Carl Roth, Cat # T143.1).

One µl of DNase I and 1 µl of 10X reaction buffer with MgCl2 (Thermo Fischer Scientific, Cat # ENO521) were added to remove any contaminating traces of genomic DNA. The samples were incubated at 37 °C for 30 min. Then, phenol-chloroform extraction was performed.

One hundred and ten µl of nuclease-free water and 200 µl of Phenol/Chloroform/Isoamyl alcohol (Carl Roth, Cat # A156.1) were added to each sample. The samples were vigorously vortexed for 1 min and centrifuged for 10 min at 12 000 g. The upper aqueous phase was removed and transferred to a PCR-clean 1.5 ml tube, then 200 µl of Chloroform were added. The samples were vortexed for 1 min and incubated at room temperature for 10 min then centrifuged for 10 min at 12 000 g. Five hundred microliters of isopropanol and 15 µl of ammonium acetate solution (Fluka, Cat # 09691-100ML) were added to the upper aqueous phase after transferring into a new tube. The samples were stored at -20 °C overnight to precipitate RNA. The samples were then centrifuged at 14 000 g for 15 min, and the pellets were washed with ethanol. Finally, the RNA pellet was resuspended in 50 µl of nuclease-free water. The concentration and quality of RNA were assessed using NanoDrop ND-1000 spectrophotometer (Peqlab).

- - 1. **cDNA synthesis (Reverse transcription)**

The RNA was reverse transcribed into library cDNA using High-capacity reverse transcription kit (Applied biosystems, Thermo Fischer Scientific, Cat # 4368814) according to manufacturer instruction. For each sample, 1000 ng of RNA were used per 20 µl reaction volume.

- - 1. **Quantitative Real-Time PCR**

To test the efficiency of gene knockdown, a quantitative real-time PCR (qRTPCR) on the transcribed cDNA of *Tenebrio molitor* fat bodies. The qRTPCR was carried out using qPCR Mastermix Plus for SYBR Green I – dTTP (Eurogentec, Cat # RT-SN2X-03+WOUN). 6.25 µl of SYBR Green master mix was mixed with 2 µl of cDNA and 0.5 µl of each of forward and reverse primers (1:10 dilution of 100 pmol/ µl, Table S2). The mixture was adjusted with nuclease-free water to a total volume of 12 µl.

The PCR plates were incubated in Eppendorf MasterCycler RealPlex (Eppendorf AG, Hamburg, Germany) for 2 min at 50 °C, followed by 10 min at 95 °C for denaturation. Then 40 PCR cycles were performed, which consisted of 15 sec denaturation at 95 °C, and 1 min annealing at 60 °C. The resulted Ct values (the cycle quantification value), which is the number of PCR cycle at which a reaction curve intersects the threshold line) were analyzed at a threshold of 200.

| Table S2: list of primers used in the current study | | | |
| --- | --- | --- | --- |
| Primer | Type | Sequence | Reference |
| Lysozyme (*Galleria mellonella)-fw* | T7 | TAA TAC GAC TCA CTA TAG GGA GAG CAA GCC GAA TAA AAA TGG A | (Khan, Agashe, & Rolff, 2017; Zanchi, Johnston, & Rolff, 2017) |
| Lysozyme (*Galleria mellonella)-rv* | T7 | TAA TAC GAC TCA CTA TAG GGA GAT ATC TGG CAG CGG CTT ATT T |  |
| Pro-phenoloxidase 1 (*T. molitor*) PO1-fw | T7 | TAA TAC GAC TCA CTA TAG GGA GAA GAG GCG TAT TTC CCC AAG | (Khan et al., 2017) |
| Pro-phenoloxidase 1 (*T. molitor*) PO1-rv | T7 | TAA TAC GAC TCA CTA TAG GGA GAG ATT CCT TCG TTC TCG GTC |  |
| Pro-phenoloxidase 2 (*T. molitor*) PO2-fw | T7 | TAA TAC GAC TCA CTA TAG GGA GAA ATT CTT GAT TCT GTA GAT |  |
| Pro-phenoloxidase 2 (*T. molitor*) PO2-rv | T7 | TAA TAC GAC TCA CTA TAG GGA GAG AGA GAT CCT GTG TTC TT |  |
| Tenecin 1 (*T. molitor*)-fw | T7 | TAA TAC GAC TCA CTA TAG GGA GAC ACG AGA TCA CGA TGA AGC | (Zanchi et al., 2017) |
| Tenecin 1 (*T. molitor*)-rv | T7 | TAA TAC GAC TCA CTA TAG GGA GAA AAT CA G TTT TTA TTT ATC GTC ATG TT |  |
| Tenecin 2 (*T. molitor*)-fw | T7 | TAA TAC GAC TCA CTA TAG GGA GAA TCA GTT CGC TTT CGA ACA GTC T |  |
| Tenecin 2 (*T. molitor*)-rv | T7 | TAA TAC GAC TCA CTA TAG GGA GAA ATT AGT TTC CAT TGA AAT GGT TTG |  |
| Tenecin 4 (*T. molitor*)-fw | T7 | TAA TAC GAC TCA CTA TAG GGA GAA TGT TAA AAG CGG TTC AAT TCG |  |
| Tenecin 4 (*T. molitor*)-rv | T7 | TAA TAC GAC TCA CTA TAG GGA GAA AAA ATC TAC TGT TCC TGT GAG GTG |  |
| RPL27A housekeeping (*T. molitor*)-fw | qPCR | TCG ACT CAT AAG AAA AAG ACA AGA AA | (Khan et al., 2017; Zanchi et al., 2017) |
| RPL27A housekeeping (*T. molitor*)-rv | qPCR | CAT TAC CGC GAC CTC CTG |  |
| Pro-phenoloxidase (*T. molitor*)-fw | qPCR | GCA CGA GCT GGA ATT GTG T | (Khan et al., 2017) |
| Pro-phenoloxidase (*T. molitor*)-rv | qPCR | GGT CGA ACA AAC AGG AGG ATG |  |
| Tenecin 1 (*T. molitor*)-fw | qPCR | GGA AGC GGC AAC AGC TGA AGA AAT | (Zanchi et al., 2017) |
| Tenecin 1 (*T. molitor*)-rv | qPCR | AAC GCA GAC CCT CTT TCC GTT ACA |  |
| Tenecin 2 (*T. molitor*)-fw | qPCR | GAA TGG AGG GTG GTC CGT CAA C |  |
| Tenecin 2 (*T. molitor*)-rv | qPCR | TTG TGC TGC ACC TCA ACG TTG GTC |  |
| Tenecin 4 (*T. molitor*)-fw | qPCR | CAA CAA CGG CGG CCA CAA ATT AGA |  |
| Tenecin 4 (*T. molitor*)-rv | qPCR | TGT AAT CCA GCT TCC CAC CGA AGA |  |

1. **Supplementary materials to the results**
   1. **Results of RNAi- based knockdown efficiency assessment**


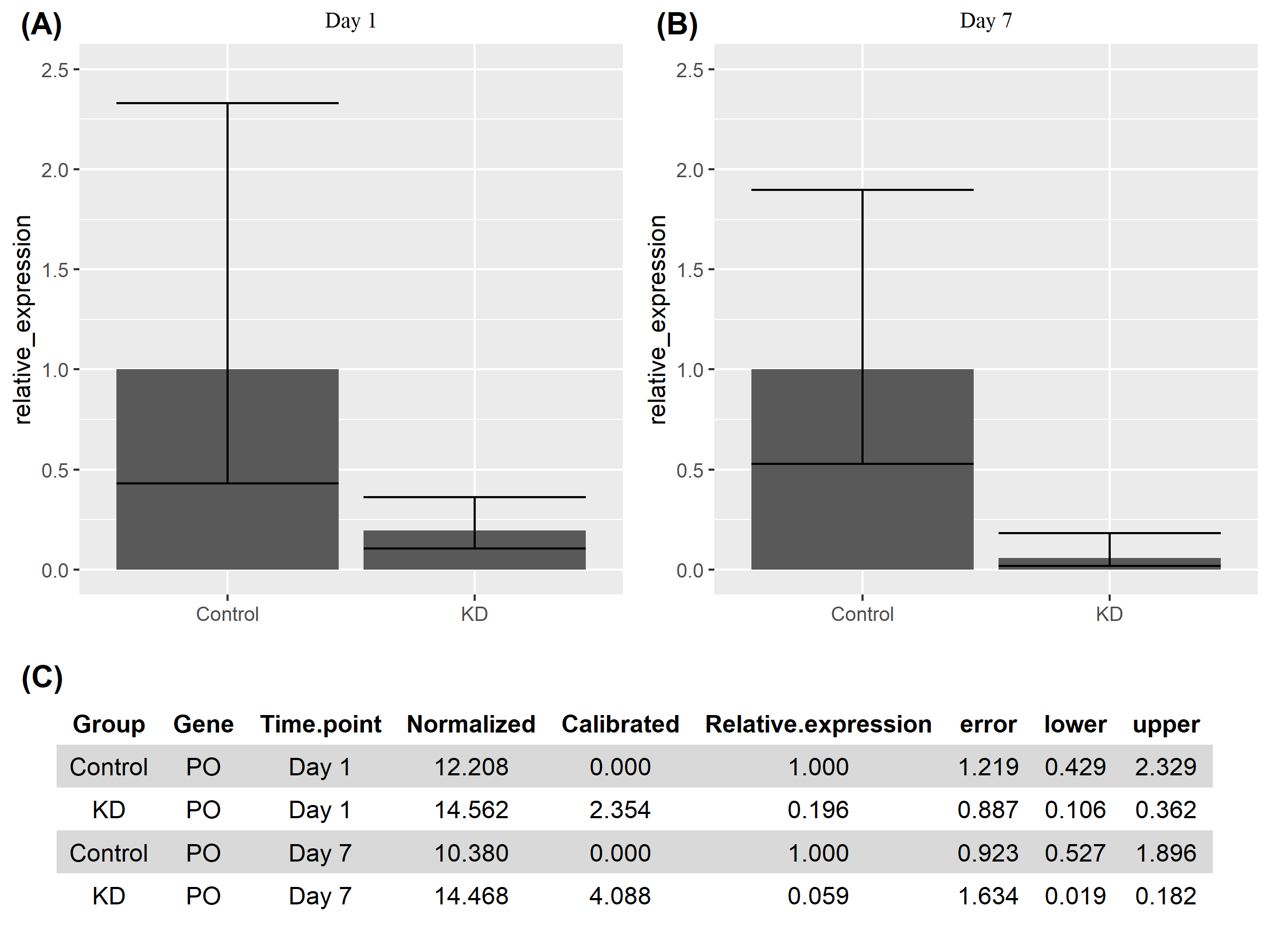


**Figure S3**: Relative expression (2^-ΔΔCT^) of the prophenoloxidase gene compared to house-keeping gene/internal control (ribosomal protein RPL27A gene) in both beetles with normal expression levels of prophenoloxidase “**Control**” and beetles with knockdown expression of prophenoloxidase “**KD**” at: **(A)** 1-day post-infection with different *S. aureus* strains (there is a significant average 80.44% reduction of relative expression of the prophenoloxidase gene in “**KD**” beetles compared to “**Control**” (log_10_ (ΔCT_PO_) 0.06 vs log_10_ (ΔCT_Control_) 1.06; F_1, 88_ = 24.99; p<0.0001)). **(B**) 7-days post-*S. aureus* injection (there is a significant average 94.1% reduction of expression of the prophenoloxidase gene in “**KD**” beetles compared to **Control** (log_10_ (ΔCT_PO_) 0.1223 vs log_10_ (ΔCT_Control_) 0.999; F_1, 88_ = 49.54; p<0.0001)). (**C**) The table summarizes the normalized (ΔCT), calibrated (ΔΔCT) and relative prophenoloxidase gene expression (2^-ΔΔCT^) ± standard error at different time points post-infection as calculated by the “pcr_analyze” function from “pcr” package in R.


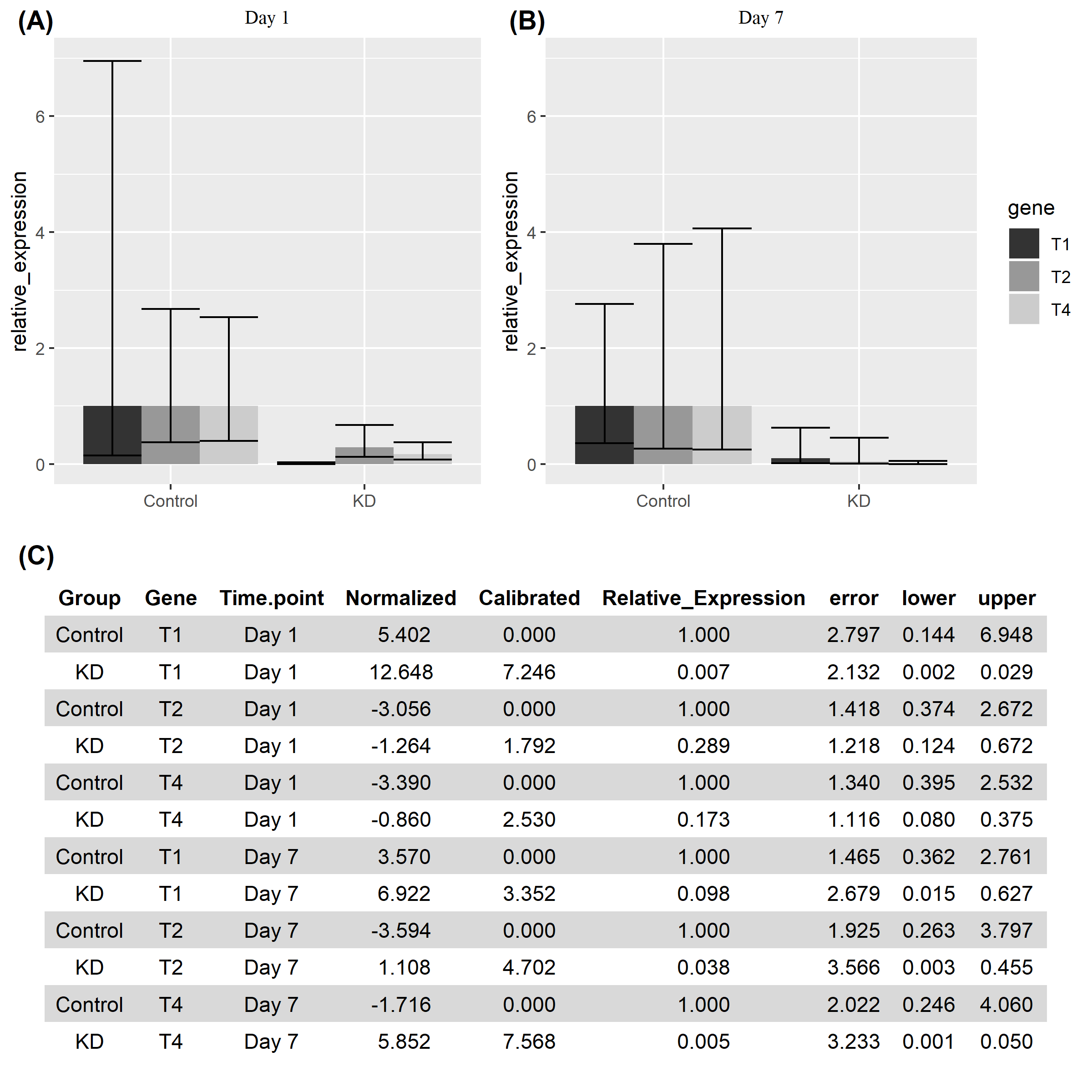
**Figure S4**: Relative expression “2^-ΔΔCT^” of tenecin 1, tenecin 2 and tenecin 4 genes, respectively, compared to house-keeping gene/internal control (ribosomal protein RPL27A gene) in both beetles with normal expression levels of AMP “**Control**” and beetles with AMP knockdown “**KD**” treatment at: **(A)** 1-day post-infection with different *S. aureus* strains (there is a significant average 99.3% reduction of relative expression of the tenecin 1 gene (log_10_ (ΔCT _T1_) 0.411 vs log_10_ (ΔCT _Control_) 0.557; F_1, 88_ = 37.13; p<0.0001), 80% reduction of relative expression of the tenecin 2 gene (log_10_ (ΔCT _T2_) 0.064 vs log_10_ (ΔCT _Control_) 1.129; F_1, 77_ = 74.06; p<0.0001), and 82.7% reduction of relative expression of the tenecin 4 (log_10_ (ΔCT _T4_) 0.064 vs log_10_ (ΔCT _Control_) 1.135; F_1, 88_ = 57.85; p<0.0001) in “**KD**” beetles compared to “**Control**” individuals. **(B**) 7-days post-*S. aureus* injection (there is a significant average 90.2% reduction of relative expression of the tenecin 1 gene (log_10_ (ΔCT _T1_ + 6) 0.245 vs log_10_ (ΔCT _Control_ + 6) 0.949; F_1, 88_ = 26.92; p<0.0001), 96.2% reduction of relative expression of the tenecin 2 gene (log_10_ (ΔCT _T2_ + 6) 0.501 vs log_10_ (ΔCT _Control_ + 6) 0.315; F_1, 88_ = 28.78; p < 0.0001), and 99.5% reduction of relative expression of the tenecin 4 (log_10_ (ΔCT _T4_ +6) 0.309 vs log_10_ (ΔCT _Control_ + 6) 0.535; F_1, 88_ = 24.98; p < 0.0001) in AMP.KD beetles compared to control individuals. (**C**) The table summarizes the normalized (ΔCT), calibrated (ΔΔCT) and comparative relative tenecin 1, tenecin 2, and tenecin 4 gene expression (2^-ΔΔCT^) ± standard error at different time points post-infection as calculated by the “pcr_analyze” function from “pcr” package in R.

- 1. **Bacterial load of the 9 studied strain in the current study at day 1 and day 7 post-infection**


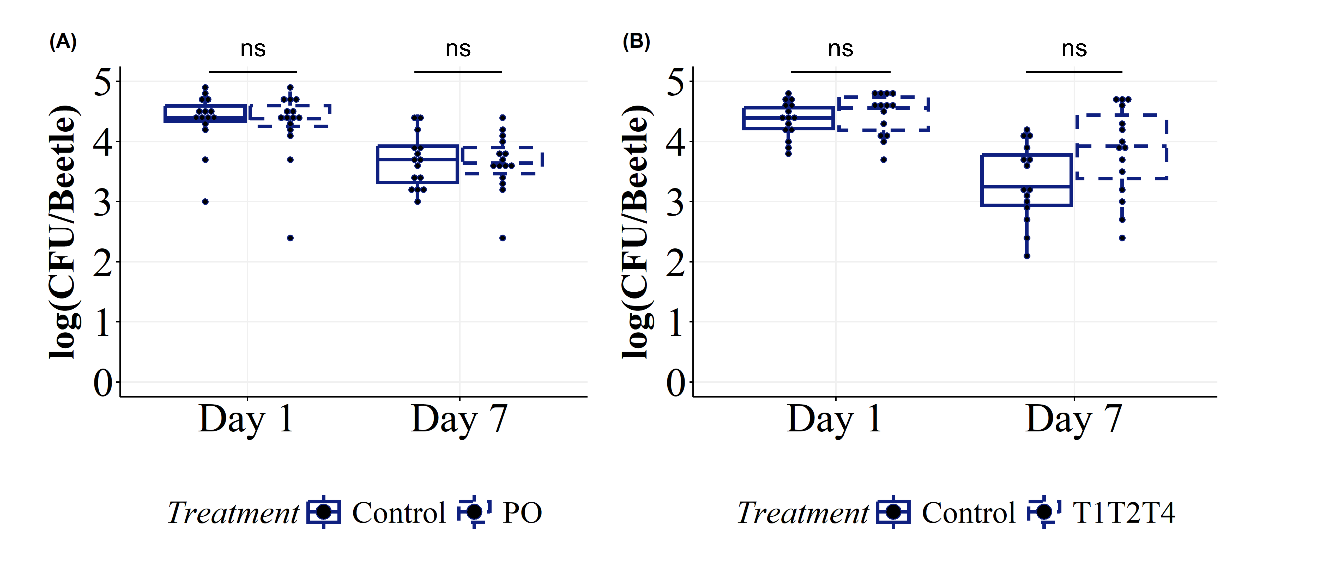


**Figure S5** (**A**-B) Bacterial load of *Staphylococcus aureus* ancestor (SH1000) strain in *Tenebrio molitor* at different time points post-infection. The colony-forming units (CFU) recovered from 100 μl of hemolymph are represented on a log scale by box plots showing quartiles and medians. The bars represent the 1.5 interquartile. **Control** (**solid outline**) refers to bacterial load of *S. aureus* in beetles which receive control RNAi. The left-hand side panel (**A**) represents bacterial load data obtained from the phenoloxidase knockdown experiment. The **PO KD** (**dotted outline**) refers to bacterial load of *S. aureus* in beetles with knocked-down expression of prophenoloxidase. The right-hand side panel (**B**) represents the bacterial load data obtained from the AMPs knockdown experiment. **AMPS KD** (**dotted outline**) refers to bacterial load of *S. aureus* in beetles with knocked-down expression of tenecin 1, tenecin 2 and tenecin 4. (n = 15 beetles/group divided into three replicates; ns: p > 0.05).

**
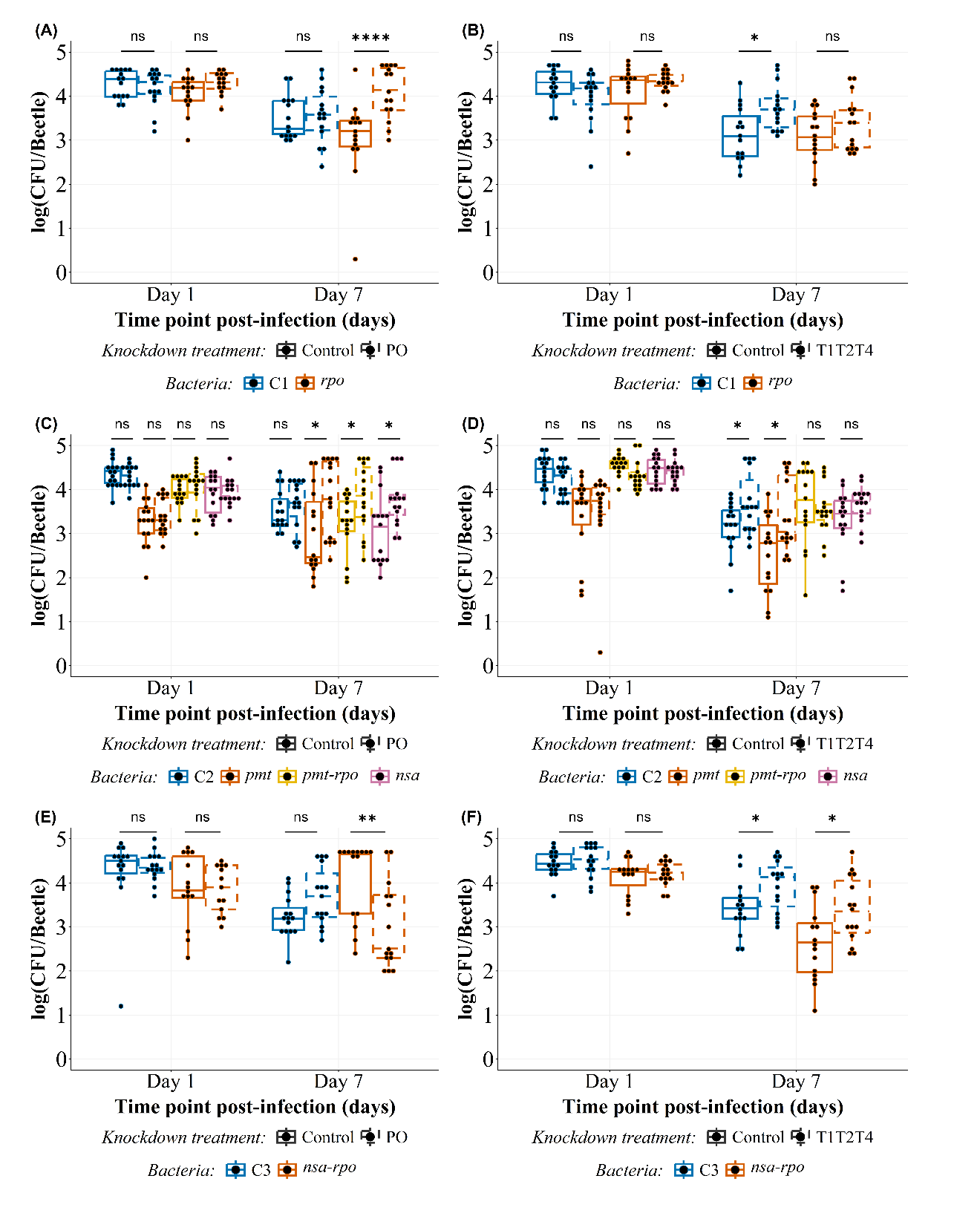
Figure S6** (**A**-**F**) Bacterial load of *Staphylococcus aureus* strain (Line 1, 2 and 3, Table 1) in *Tenebrio molitor* at different time point post-infection. The colony-forming units (CFU) recovered from 100 μl of hemolymph are represented on a log scale by box plots showing quartiles and medians. The bars represent the 1.5 interquartile. **Control** (**solid outline**) refers to bacterial load of *S. aureus* in beetles which receive control RNAi. The left-hand side panels (**A, C and E**) represent bacterial load data obtained from the phenoloxidase knockdown experiment. The **PO KD** (**dotted outline**) refers to bacterial load of *S. aureus* in beetles with knocked-down expression of prophenoloxidase. The right-hand side panels (**B, D and F**) represent the bacterial load data obtained from the AMPs knockdown experiment. **AMPS KD** (**dotted outline**) refers to bacterial load of *S. aureus* in beetles with knocked-down expression of tenecin 1, tenecin 2 and tenecin 4. (n = 15 beetles/group divided into three replicates; ns: p > 0.05, *: p < 0.05, **: p < 0.01 and ****: p ≤ 0.0001).

**3.3. Pairwise comparison stats of bacterial load of the 9 *S. aureus* strains studied herein.**

**Table S3** Comparison of CFU of *Staphylococcus aureus* strains recovered from beetles with PO knock-down or triple AMP knock-down treatments to their procedural control beetles using effect sizes. The bacterial load is assessed for each strain at 2 time points post-infection (Day 1 and Day 7). The data is plotted in Fig. S7.

| **Mutants** | **KD** | **Day** | | **hedges** | **low hedge** | **high hedge** | **Cohen's d** | | **Low CI** | **High CI** |
| --- | --- | --- | --- | --- | --- | --- | --- | --- | --- | --- |
| *rpo* | PO | | 1 | -0.54 | -1.24 | 0.17 | -0.57 | -1.34 | | 0.19 |
| *pmt* | PO | | 1 | -0.17 | -0.87 | 0.53 | -0.17 | -0.92 | | 0.57 |
| *pmt-rpo* | PO | | 1 | -0.57 | -1.28 | 0.15 | -0.6 | -1.36 | | 0.17 |
| *nsa* | PO | | 1 | 0.33 | -0.37 | 1.02 | 0.34 | -0.41 | | 1.09 |
| *nsa-rpo* | PO | | 1 | 0.03 | -0.66 | 0.73 | 0.033 | -0.72 | | 0.78 |
| A | PO | | 1 | 0.1 | -0.6 | 0.79 | 0.1 | -0.65 | | 0.85 |
| C1 | PO | | 1 | 0.24 | -0.47 | 0.93 | 0.24 | -0.51 | | -0.5 |
| C2 | PO | | 1 | 0.29 | -0.41 | 0.98 | 0.3 | -0.46 | | 1.05 |
| C3 | PO | | 1 | 0.14 | -0.56 | 0.84 | 0.15 | -0.6 | | 0.89 |
| *rpo* | PO | | 7 | -1.16 | -1.93 | -0.37 | -1.21 | -2.02 | | -0.4 |
| *pmt* | PO | | 7 | -0.67 | -1.38 | 0.05 | -0.69 | -1.46 | | 0.08 |
| *pmt-rpo* | PO | | 7 | -0.95 | -1.7 | -0.18 | -1 | -1.8 | | -0.21 |
| *nsa* | PO | | 7 | -0.49 | -1.19 | 0.22 | -0.51 | -1.27 | | 0.25 |
| *nsa-rpo* | PO | | 7 | 1.15 | 0.38 | 1.9 | 1.18 | 0.37 | | 1.99 |
| A | PO | | 7 | 0.11 | -0.59 | 0.8 | 0.11 | -0.63 | | 0.86 |
| C1 | PO | | 7 | -0.17 | -0.86 | 0.53 | -0.17 | -0.92 | | 0.57 |
| C2 | PO | | 7 | -0.21 | -0.9 | 0.49 | -0.21 | -0.96 | | 0.53 |
| C3 | PO | | 7 | -0.79 | -1.51 | -0.05 | -0.83 | -1.61 | | -0.05 |
| *rpo* | T1T2T4 | | 1 | -0.09 | -0.78 | 0.6 | -0.09 | -0.84 | | 0.65 |
| *pmt* | T1T2T4 | | 1 | -0.28 | -0.96 | 0.41 | -0.29 | -1.04 | | 0.46 |
| *pmt-rpo* | T1T2T4 | | 1 | 0.42 | -0.29 | 1.12 | 0.43 | -0.32 | | 1.19 |
| *nsa* | T1T2T4 | | 1 | 0.17 | -0.53 | 0.86 | 0.17 | -0.58 | | 0.92 |
| *nsa-rpo* | T1T2T4 | | 1 | -0.04 | -0.74 | 0.65 | -0.04 | -0.79 | | 0.7 |
| A | T1T2T4 | | 1 | 0.12 | -0.13 | 0.37 | 0.12 | -0.13 | | 0.37 |
| C1 | T1T2T4 | | 1 | 0.59 | -0.13 | 1.3 | 0.61 | -0.15 | | 1.37 |
| C2 | T1T2T4 | | 1 | 0.66 | -0.07 | 1.37 | 0.67 | -0.09 | | 1.45 |
| C3 | T1T2T4 | | 1 | -0.36 | -1.06 | 0.35 | -0.37 | -1.12 | | 0.38 |
| *rpo* | T1T2T4 | | 7 | -0.53 | -1.23 | 0.18 | -0.56 | -1.32 | | 0.2 |
| *pmt* | T1T2T4 | | 7 | -0.81 | -1.54 | -0.06 | -0.86 | -1.64 | | -0.08 |
| *pmt-rpo* | T1T2T4 | | 7 | 0.5 | -0.21 | 1.2 | 0.52 | -0.24 | | 1.28 |
| *nsa* | T1T2T4 | | 7 | -0.47 | -1.17 | 0.24 | -0.49 | -1.24 | | 0.28 |
| *nsa-rpo* | T1T2T4 | | 7 | -0.65 | -1.35 | 0.08 | -0.68 | -1.45 | | 0.09 |
| A | T1T2T4 | | 7 | -0.5 | -0.75 | -0.24 | -0.5 | -0.76 | | -0.24 |
| C1 | T1T2T4 | | 7 | -0.65 | -1.36 | 0.08 | -0.68 | -1.45 | | 0.09 |
| C2 | T1T2T4 | | 7 | -0.77 | -1.5 | -0.03 | -0.82 | -1.6 | | -0.04 |
| C3 | T1T2T4 | | 7 | -0.69 | -1.4 | 0.04 | -0.71 | -1.48 | | 0.06 |


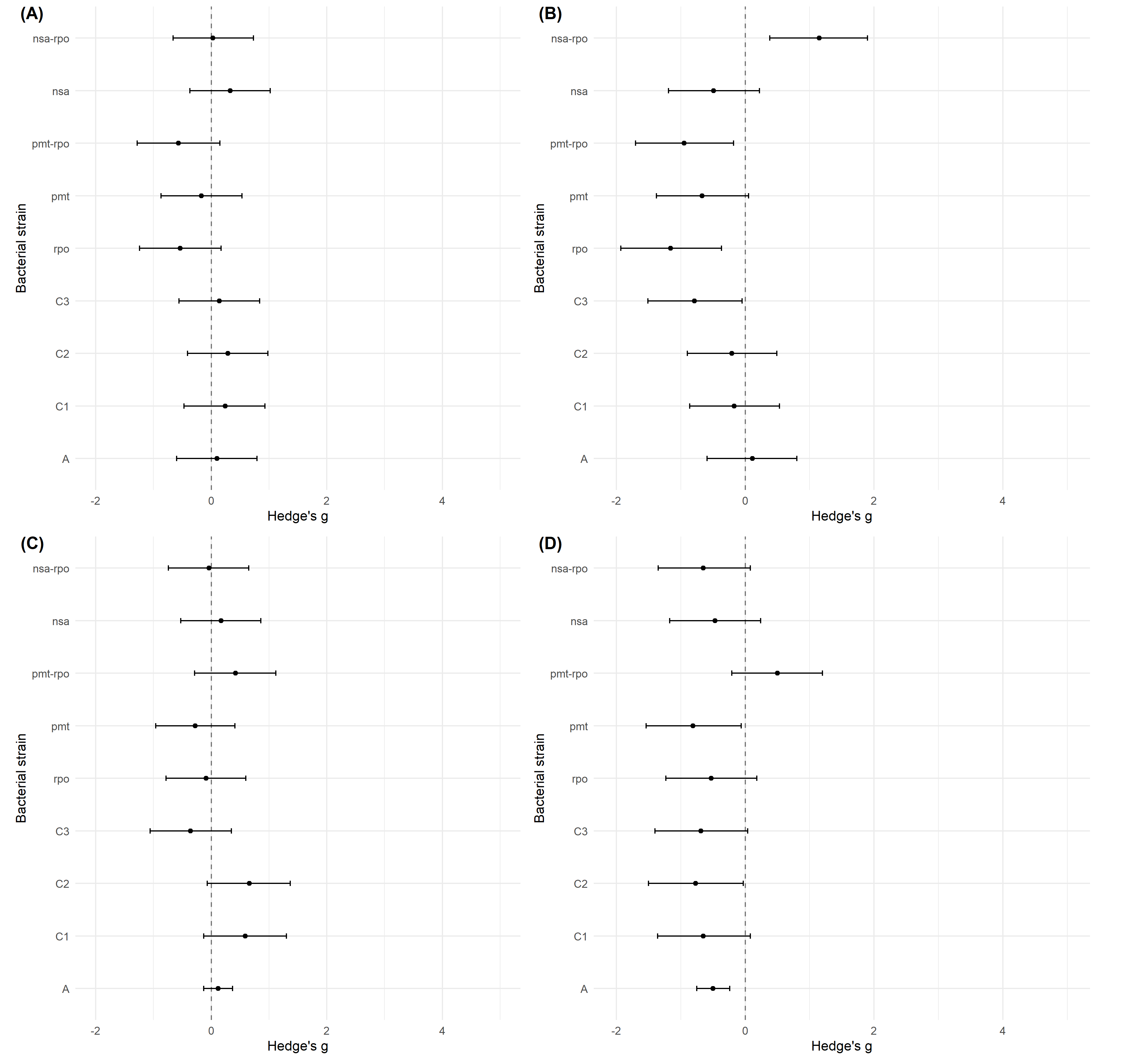


**Figure S7.** (**A-D**) Hedge’s g as a measure of effect sizes of colony forming units of tenecin-sensitive and tenecin-resistant *S. aureus* strains recovered from: (**A**) beetles with PO knockdown treatment one day post infection. (**B**) beetles with PO knockdown treatment seven days post infection. (**C**) beetles with tenecin 1, 2 and 4 knockdown treatment one day post infection. (**D**) beetles with tenecin 1, 2 and 4 knockdown treatment seven days post infection.

1. **References:**

Arii, K., Kawada-Matsuo, M., Oogai, Y., Noguchi, K., & Komatsuzawa, H. (2019). Single mutations in BraRS confer high resistance against nisin A in *Staphylococcus aureus*. *MicrobiologyOpen, 8*(11), e791. doi:10.1002/mbo3.791

Cheung, G. Y. C., Fisher, E. L., McCausland, J. W., Choi, J., Collins, J. W. M., Dickey, S. W., & Otto, M. (2018). Antimicrobial Peptide Resistance Mechanism Contributes to *Staphylococcus aureus* Infection. *J Infect Dis, 217*(7), 1153-1159. doi:10.1093/infdis/jiy024

Cui, L., Isii, T., Fukuda, M., Ochiai, T., Neoh, H.-m., Camargo, I. L. B. d. C., . . . Hiramatsu, K. (2010). An RpoB Mutation Confers Dual Heteroresistance to Daptomycin and Vancomycin in *Staphylococcus aureus*. *Antimicrobial agents and chemotherapy, 54*(12), 5222-5233. doi:doi:10.1128/AAC.00437-10

El Shazely, B., Urbanski, A., Johnston, P. R., & Rolff, J. (2019). In vivo exposure of insect AMP resistant *Staphylococcus aureus* to an insect immune system. *Insect Biochem Mol Biol, 110*, 60-68. doi:10.1016/j.ibmb.2019.04.017

Gómez Casanova, N., Siller Ruiz, M., & Muñoz Bellido, J. L. (2017). Mechanisms of resistance to daptomycin in *Staphylococcus aureus*. *Rev Esp Quimioter, 30*(6), 391-396.

Hiron, A., Falord, M., Valle, J., Débarbouillé, M., & Msadek, T. (2011). Bacitracin and nisin resistance in *Staphylococcus aureus*: a novel pathway involving the BraS/BraR two‐component system (SA2417/SA2418) and both the BraD/BraE and VraD/VraE ABC transporters. *Molecular microbiology, 81*(3), 602-622. doi:10.1111/j.1365-2958.2011.07735.x

Joo, H.-S., Fu, C. I., & Otto, M. (2016). Bacterial strategies of resistance to antimicrobial peptides. *Philosophical transactions of the Royal Society of London. Series B, Biological sciences, 371*(1695), 20150292. doi:10.1098/rstb.2015.0292

Kawada-Matsuo, M., Le, M. N.-T., & Komatsuzawa, H. (2021). Antibacterial Peptides Resistance in *Staphylococcus aureus*: Various Mechanisms and the Association with Pathogenicity. *Genes, 12*(10), 1527.

Kawada-Matsuo, M., Oogai, Y., Zendo, T., Nagao, J., Shibata, Y., Yamashita, Y., . . . Komatsuzawa, H. (2013). Involvement of the novel two-component NsrRS and LcrRS systems in distinct resistance pathways against nisin A and nukacin ISK-1 in *Streptococcus* mutans. *Applied and Environmental Microbiology, 79*(15), 4751-4755. doi:10.1128/AEM.00780-13

Khan, I., Agashe, D., & Rolff, J. (2017). Early-life inflammation, immune response and ageing. *Proceedings of the Royal Society B: Biological Sciences, 284*(1850), 20170125. doi:10.1098/rspb.2017.0125

Lee, Y. H., Nam, K. H., & Helmann, J. D. (2013). A mutation of the RNA polymerase beta' subunit (rpoC) confers cephalosporin resistance in *Bacillus subtilis*. *Antimicrob Agents Chemother, 57*(1), 56-65. doi:10.1128/AAC.01449-12

Li, M., Cha, D. J., Lai, Y., Villaruz, A. E., Sturdevant, D. E., & Otto, M. (2007). The antimicrobial peptide-sensing system aps of *Staphylococcus aureus*. *Mol Microbiol, 66*(5), 1136-1147. doi:10.1111/j.1365-2958.2007.05986.x

Ma, Z., Lasek-Nesselquist, E., Lu, J., Schneider, R., Shah, R., Oliva, G., . . . Malik, M. (2018). Characterization of genetic changes associated with daptomycin nonsusceptibility in *Staphylococcus aureus*. *PloS one, 13*(6), e0198366. doi:10.1371/journal.pone.0198366

Makarova, O., Johnston, P., Rodriguez-Rojas, A., El Shazely, B., Morales, J. M., & Rolff, J. (2018). Genomics of experimental adaptation of *Staphylococcus aureus* to a natural combination of insect antimicrobial peptides. *Sci Rep, 8*(1), 15359. doi:10.1038/s41598-018-33593-7

Peschel, A., & Otto, M. (2013). Phenol-soluble modulins and staphylococcal infection. *Nature Reviews Microbiology, 11*(10), 667-673. doi:10.1038/nrmicro3110

Yoshida, Y., Matsuo, M., Oogai, Y., Kato, F., Nakamura, N., Sugai, M., & Komatsuzawa, H. (2011). Bacitracin sensing and resistance in *Staphylococcus aureus*. *FEMS Microbiol Lett, 320*(1), 33-39. doi:10.1111/j.1574-6968.2011.02291.x

Zalenskaya, K., Lee, J., Gujuluva, C. N., Shin, Y. K., Slutsky, M., & Goldfarb, A. (1990). Recombinant RNA polymerase: inducible overexpression, purification and assembly of *Escherichia coli rpo* gene products. *Gene, 89*(1), 7-12. doi:10.1016/0378-1119(90)90199-2

Zanchi, C., Johnston, P. R., & Rolff, J. (2017). Evolution of defence cocktails: Antimicrobial peptide combinations reduce mortality and persistent infection. *Mol Ecol, 26*(19), 5334-5343. doi:10.1111/mec.14267
