# Supplementary figures and images for: A Trade‐Off Between Antimicrobial Peptide Resistance and Sensitivity to Host Immune Effectors in Staphylococcus aureus In Vivo
Source: Evol Appl. 2025 Feb 6;18(2):e70068. doi: 10.1111/eva.70068 (PMC11802329; doi:10.1111/eva.70068)

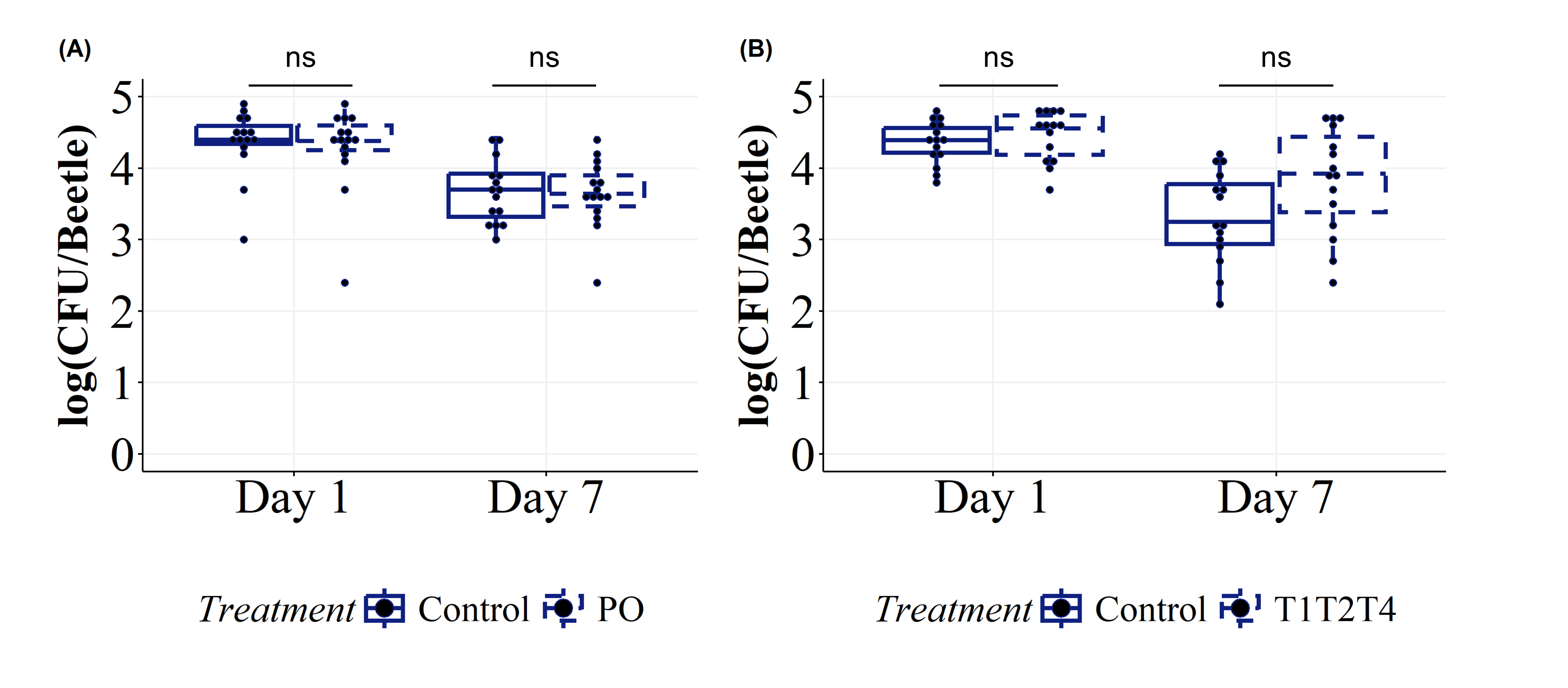

Supplement: Supplementary file 1 — Appendix S1: [file EVA-18-e70068-s001.zip › fig s5.tif]

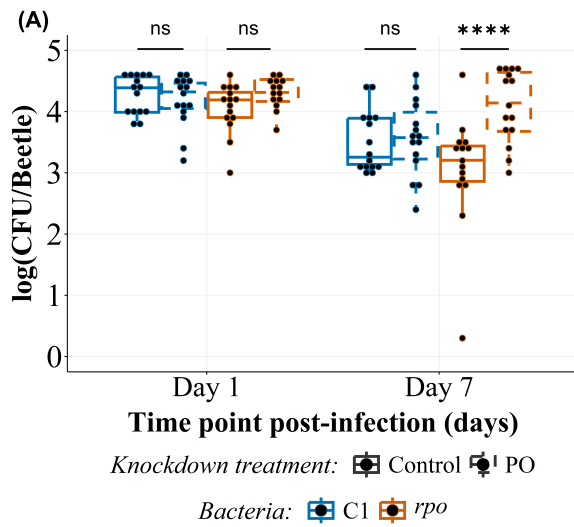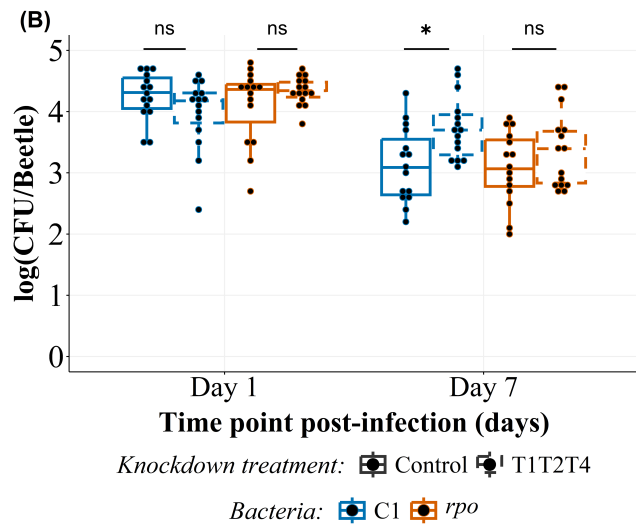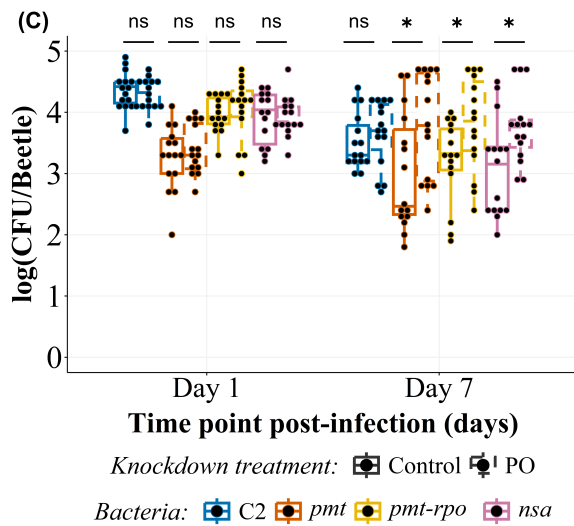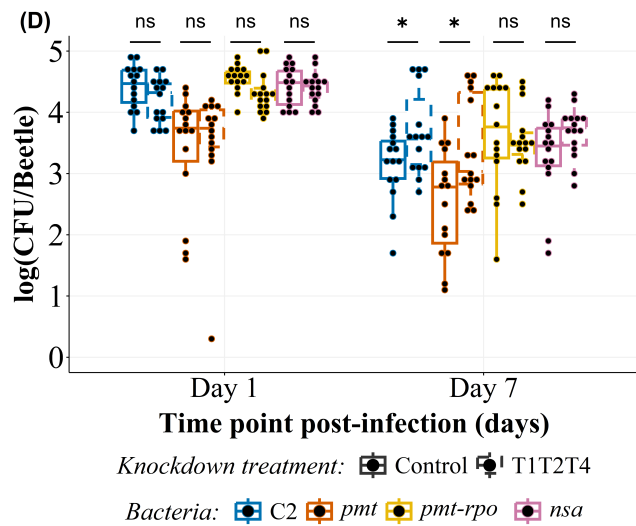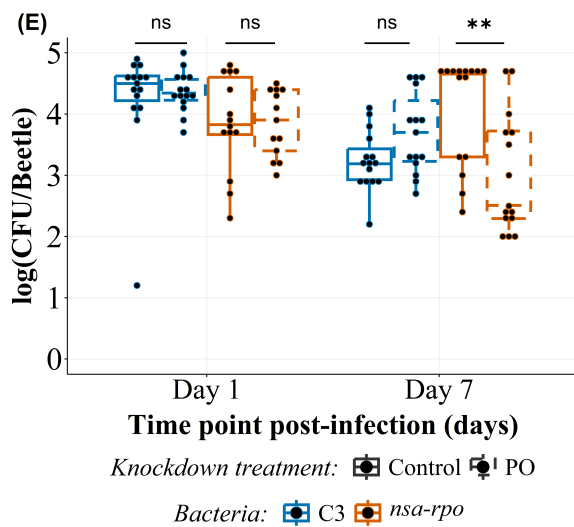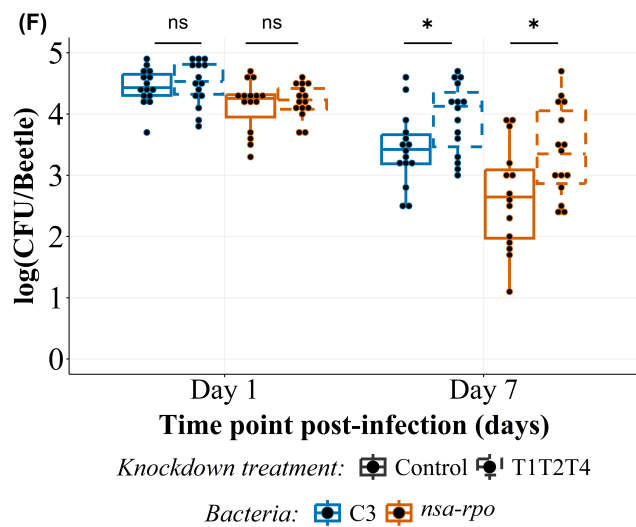

Supplement: Supplementary file 1 — Appendix S1: [file EVA-18-e70068-s001.zip › fig s6.pdf]
